# Islet Amyloid Polypeptide Modelled to Simulate Diabetes Co‐Oligomerized with β‐Amyloid 1‐42 Reproducing the Pathological Cascade of Alzheimer's Disease in Human Cerebral Organoids

**DOI:** 10.1002/advs.202516837

**Published:** 2026-01-22

**Authors:** Jin Yan, Zhimeng Tang, Yanyu Luo, Bin Liu, Xinxin Huang, Jun Wei, Feng Yue

**Affiliations:** ^1^ State Key Laboratory of Digital Medical Engineering School of Biomedical Engineering Hainan University Sanya China; ^2^ Collaborative Innovation Center of One Health Hainan University Haikou China; ^3^ iRegene Therapeutics Co. Ltd. Chengdu China

**Keywords:** Alzheimer's disease, human cerebral organoids, β‐amyloid oligomers, neuronal death, Type 2 diabetes mellitus, islet amyloid polypeptide

## Abstract

Previous animal models of sporadic Alzheimer's disease (sAD), based on the β‐amyloid (Aβ) cascade hypothesis and induced by Aβ1‐42 oligomers (AβO), only recapitulated early AD pathological features. sAD cerebral organoids (COs) model employed Aβ coculture approach and found no typical features of AD pathology. Type 2 diabetes (T2DM) is one of the most important modifiable AD risk factors, so we hypothesize that T2DM could substantially exacerbate Aβ neurotoxicity and reproduce typical AD pathology. Human islet amyloid polypeptide (hIAPP) was used to mimic T2DM and was co‐oligomerized with Aβ1‐42 peptide, and delivered to the central of human iPSC‐derived mature COs through intermittently repeated microinjections, so as to simulate the chronic exposure to Aβ within the brain. The Aβ42‐hIAPP co‐oligomers induced a pathological phenotype more closely resembling the pathological features of advanced AD, notably, neuronal density showed significant reduction, with 3.2 times more neuronal death. Dynamic metabolomic analysis revealed the metabolic pathways and differential metabolites that may be correlated to the primary mechanism underlying the enhanced neurotoxic effects and accelerated AD pathology. Furthermore, this study developed a sAD CO model more resembling the pathological features of advanced AD, which potentially provides a valuable platform for AD pathogenesis research and novel drug screening.

## Graphical abstract

Human islet amyloid polypeptide (hIAPP) was used to mimic T2DM, and Aβ42‐hIAPP co‐oligomers were delivered into the human mature cerebral organoids (COs), which reproduce typical AD pathology and significant neuronal death more closely resembling that of AD patients. This suggests T2DM may be involved in the Aβ cascade hypothesis and provides a valuable sporadic AD COs model for translational research.

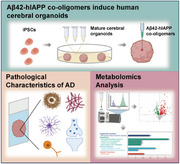

## 1 Introduction

Alzheimer's disease (AD) is the most common form of dementia. According to the World Alzheimer Report 2025, the global number of dementia patients is projected to rise from the current 55 to 139 million by 2050 [1]. AD is an age‐related neurodegenerative disorder that initially presents with mild memory impairment and progressively affects behavior, language, and judgement, ultimately leading to severe cognitive dysfunction [2]. The complete AD pathology includes extracellular beta‐amyloid protein (Aβ) deposition forming senile plaques and intracellular accumulation of hyperphosphorylated tau protein (p‐tau) forming neurofibrillary tangles (NFTs). These features are accompanied by dystrophic synapses, along with activation of astrocytes and microglia. Disease progression results in advanced pathology characterized by synaptic damage, neuronal degeneration, and death [3, 4, 5, 6]. Clinically, AD manifests as two types: familial AD (fAD) and sporadic AD (sAD). sAD usually develops after the age of 65 and accounts for about 95% of AD overall [7].

The lack of sAD models is a major obstacle for AD prevention and treatment. Currently, rodent‐based fAD models are primarily developed through AD‐related gene editing. However, due to inherent species differences, rodent models struggle to replicate the complexity and diversity of pathological and behavioral manifestations observed in AD patients, significantly limiting their translational research applications [8]. The construction of sAD animal model has predominantly followed the Aβ hypothesis [9, 10], employing AβO, the core components of senile plaques [11]. For instance, bilateral ventricular Aβ42 injections in rats induced Aβ deposition restricted to hippocampal regions, accompanied solely by a spatial memory deficit in behavioral tests [12]. Subsequent research administered AβO injections into monkey brains, which only recapitulated early AD pathological features [13], including typical Aβ plaques, NFTs, glial cell activation, and synaptic reduction in the monkey brains, it failed to produce advanced AD pathological features, including ghost tangles (the highest level of NFT maturity) and neuron death. Notably, cognitive memory behavior was not evaluated. Nevertheless, intracerebral injection remains to be regarded as an effective pathological modelling strategy, as it mimics chronic exposure to Aβ in the brain, as the Aβ cascade hypothesis posits that the diffusion of Aβ within the brain is a key factor driving the pathology of AD.

In recent years, human‐induced pluripotent stem cell (iPSC)‐derived 3D cerebral organoids (COs), which exhibit cellular and structural compositions resembling the human brain, have emerged as a promising platform for AD research [14]. The fAD COs based on APOE [15], APP, and/or PSEN1 [16] gene mutations have confirmed that these COs can characterize partial pathological features of AD, such as Aβ plaques and tau. In studies of sAD CO, Bejoy J et al. cocultured hiPSC‐derived cortical neuronal spheroids with AβO, observing Aβ deposition and limited p‐tau expression, though neuronal death was not characterized [17]. Another study induced Aβ accumulation, p‐tau expression, and synaptic loss in sAD CO through serum exposure, yet similarly did not assess neuronal death [18]. These studies indicate that whether AβO is injected into animal brains or cocultured with COs, it can reproduce the pathological phenotype of early AD but fails to replicate more advanced pathological features. In conclusion, the more advanced pathological features of AD are not caused solely by AβO alone, but likely result from multiple factors collectively driving the Aβ cascade.

Accumulating epidemiological evidence indicates that AD progression is influenced by multiple interacting factors. The 2024 Lancet Commission report identified type 2 diabetes mellitus (T2DM), obesity, and hypertension as important modifiable risk factors for AD, and interventions targeting these risk factors could prevent or delay nearly half of all dementia cases [19, 20, 21]. Epidemiological studies indicate T2DM increases AD risk by 1.25–1.91 times [22], leading to AD being termed type 3 diabetes [21, 23]. Clinical studies revealed that a pathologic T2DM marker, pancreatic amyloid peptide (IAPP), appears in AD patients’ brains and co‐deposited with Aβ [24]. In vitro studies have found that IAPP can cross‐seed with Aβ and produce enhanced neurotoxic effects [25]. Recent cellular experiments revealed that the Aβ42‐hIAPP co‐oligomer formed by Aβ42 and IAPP produced more severe neurotoxic effects and AD pathological in glutamatergic neurons compared to AβO alone [26]. These studies further clarify that T2DM is an important driver of AD pathogenesis. Based on these findings, further studies are needed to examine whether T2DM can simultaneously enhance Aβ‐induced neurotoxicity and reproduce typical AD pathology.

In summary, this study aimed to investigate whether Aβ neurotoxicity could be enhanced under T2DM conditions, a major AD risk factor, to induce AD cascade pathology and recapitulate the AD pathological features. Accordingly, we used hIAPP to model T2DM by forming Aβ42‐hIAPP co‐oligomerized heterocomplexes with Aβ1‐42 peptide in vitro. The Aβ42‐hIAPP co‐oligomers, along with controls (AβO, SAβ, and HEPES buffer) were intermittently and repeatedly microinjected into the centers of mature COs (100‐day cultured) using a micro‐syringe pump so as to simulate the chronic exposure conditions. Injected COs were maintained for 90 additional days before OCT embedding for histopathological assessment of AD pathology and neurotoxicity. Concurrently, CO cultures were collected pre‐injection and at 15, 45, and 90 days post‐injection of Aβ42‐hIAPP co‐oligomers, AβO, SAβ, and HEPES buffer for dynamic metabolomics analysis. This study provides a feasible approach to construct a simulated clinical AD NHP model. Additionally, we established a sAD COs model encompassing more advanced pathological features of AD, offering a valuable platform for AD pathogenesis research and novel drug target screening.

## 2 Results

### 2.1 Cultivation and Characterization of Mature COs

**FIGURE 1:**
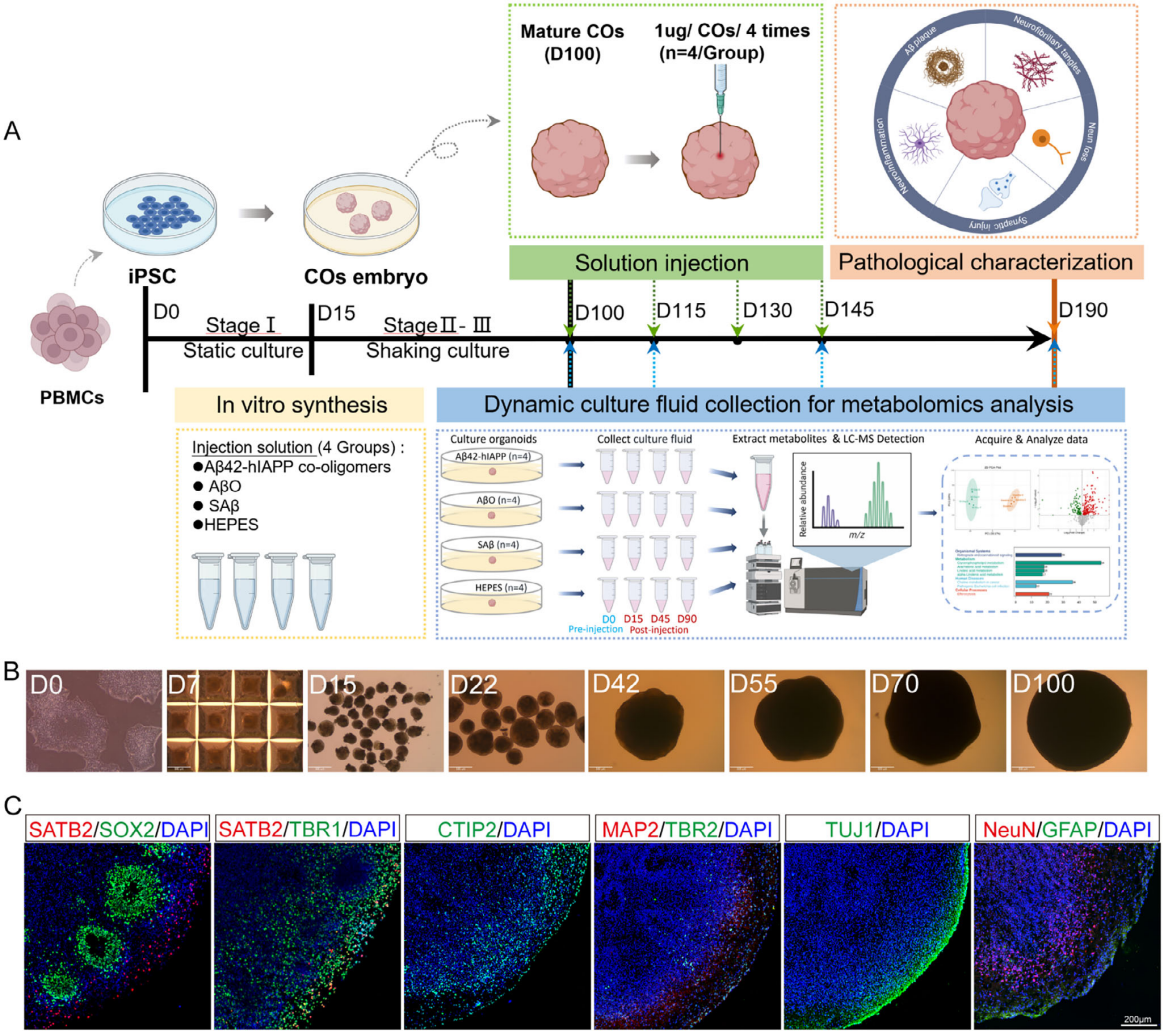
Characterization of mature COs derived from hiPSCs. A. Schematic overview of mature CO culture and experimental induction. PBMCs were first reprogrammed into iPSCs, then differentiated into mature COs (D100) through a three‐stage culture protocol. Aβ42‐hIAPP co‐oligomers, AβO and SAβ were synthesized in vitro alongside HEPES buffer preparation (yellow module). Aβ42‐hIAPP co‐oligomers, AβO, SAβ, and HEPES were injected into the center of mature COs, with four injections (1 µL each at 1 µg/µL) administered to each COs at 15‐day intervals (*n* = 4 COs per group; green module and arrow). COs culture was dynamically collected during induction (D0, D15, D45, D90) for metabolomic analysis (blue module and arrow). Histopathological examination of COs was conducted at the culture endpoint (D190; orange module and arrow). Image created with BioRender.com, with permission. Scale bar: 200 µm. B. Bright‐field microscopy of CO development from iPSCs to maturity. Scale bar: 500 µm. C. Immunofluorescence staining analysis of ventricular and neuronal composition in mature COs. SATB2: superficial cortical layer marker; TBR1 and CTIP2: deep cortical layer marker; MAP2: neuronal marker; SOX2: neural progenitor cell marker; TUJ1: neuronal marker; TBR2: intermediate progenitor marker; NeuN: neuronal marker; GFAP: astrocyte marker. Scale bar: 200 µm.

In this study, mature COs cultured via hiPSC differentiation expressed multiple ventricular markers, including the ventricular/subventricular‐like zone (VZ/SVZ) neural progenitor marker SOX2, the neuronal marker TUJ1, the superficial cortical layer marker SATB2, the deep cortical layer marker TBR1 and CTIP2, and the intermediate progenitor marker TBR2 (Figure 1C). Consistent with previous studies [27, 28], this expression profile suggests that these organoids contain cortical regions resembling those of the human brain. The quantified proportion of SOX2, TBR2, and CTIP2‐positive cells was also consistent with previous studies (Figure S1A). Mature COs also exhibited expression of other neuronal markers (Figure 1C), such as the neuronal marker MAP2, the astrocyte marker glial fibrillary acidic protein (GFAP), which displayed mature morphology with elongated protrusions, and mature neuronal nuclei (NeuN). These findings indicate that hiPSCs successfully differentiate into organoids with cortex‐like organization. Furthermore, the presence of cell types associated with AD supports their utility as a model system for studying sAD pathology. TUNEL staining revealed that cell death rates in both the internal and peripheral regions of mature COs were approximately 20% (Figure S1B,C). Notably, microglia (detected via Iba staining) were absent in the COs (data not shown).

### 2.2 In Vitro Synthesized and Characterized Aβ42‐hIAPP Co‐Oligomers were Injected into Mature CO Centers

**FIGURE 2:**
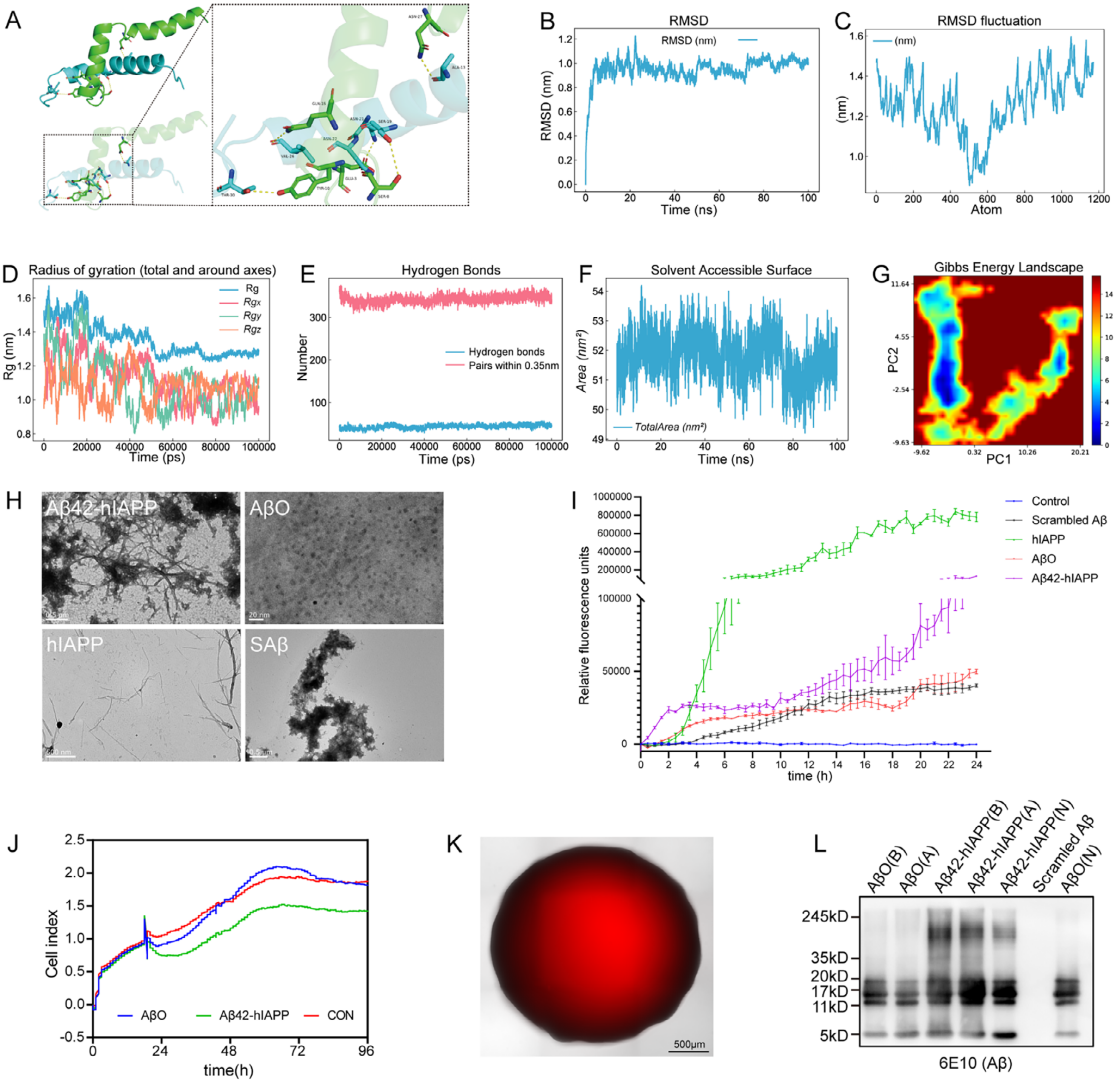
**In vitro synthesis of Aβ42‐hIAPP co‐oligomers and injection of mature CO centers**. A. Three‐dimensional representation of the optimal docking pose and interfacial residue interactions (Aβ42 in cyan; IAPP in green; hydrogen bonds shown as dashed lines). B. Root mean square deviation (RMSD) analysis results of protein complexes during the simulation process. C. Root mean square fluctuation (RMSF) values (nm) of all protein atoms. D. Radius of gyration (Rg) values (nm). E. Dynamics of the number of inter‐protein hydrogen bonds during the 100 ns simulation. F. Time evolution of the solvent accessible surface area (SASA) of the complex over 100 ns (nm^2^). G. Free energy landscape derived from principal component analysis (PCA), plotted along PC1 and PC2; the color scale (kcal/mol) denotes Gibbs free energy, with blue regions indicating low‐energy basins and red regions high‐energy states. H. The morphology of Aβ42‐hIAPP co‐oligomer, AβO, hIAPP, and SAβ was observed by Transmission electron microscopy (TEM) before injection. Scale bar: 0.5 µm, 20 nm, 500 nm, 0.5 µm. I. Aggregation kinetics of the control, scrambled Aβ, AβO, hIAPP, and Aβ42‐hIAPP co‐oligomers (10 µm) assessed by ThT fluorescence over 24 h. J. Dynamic killing process with AβOs and Aβ42‐hIAPP in SH‐SY5Y cells by smart cell real‐time monitor. K. Fluorescent imaging of injected Aβ42‐hIAPP co‐oligomers, AβO, SAβ, and HEPES solution was used pre‐injection, and fluorescent imaging was performed post‐injection to ensure that the peptide was successfully injected into the center of COs and diffused. Scale bar: 500 µm. L. Western blot analysis of Aβ42‐hIAPP co‐oligomers, AβO, SAβ before and after injection into COs centers. Aβ42‐hIAPP co‐oligomers exhibited multiple aggregate states: monomers (4.5 kD), dimers (9 kD), trimers (13.5 kD), tetramers (17 kD), intermediate oligomers (4 kD–20 kD), and high‐molecular‐weight aggregates (> 20 kD). AβOs primarily showed monomers, trimers, and tetramers. A: post injection; B: pre injection; N: newly synthesized.

In this study, molecular docking and molecular dynamics simulations were performed to analyze the interaction of IAPP with Aβ42 protein. The molecular docking of IAPP with Aβ42 revealed that the two proteins engage in extensive hydrogen bonding and electrostatic interactions at the interface, with non‐covalent contacts centered on key residues such as SER‐8, TYR‐10, GLN‐15, ASN‐27, and GLU‐3 stabilizing the complex (Figure 2A). The RMSD of the complex remained stable during the 100 ns simulation, with a fluctuation amplitude <0.4 nm (Figure 2B). RMSF analysis (Figure 2C) reveals that the overall structure of the protein complex remains stable, while atoms 400–600 exhibit significant fluctuations, suggesting inherent flexibility in this region. Rg analysis (Figure 2D) confirms structural stability: the Rg value converged to 1.30 ± 0.03 nm during the later stages of the simulation, indicating dynamic equilibrium of conformational compactness. Hydrogen bond analysis (Figure 2E) shows the count consistently maintained at 48–52 bonds, indicating stable non‐covalent interactions within the complex. During the simulation, the overall SASA fluctuated stably between 50–54 nm^2^, with reduced interfacial exposure beneficial for hydrophobic interactions (Figure 2F). The protein complex reaches its energy minimum when PC1 is within −5.66–−3.82, and PC2 is within −4.31–−2.54 (Figure 2G). In summary, the protein complex undergoes rapid conformational adjustment during the initial phase of the simulation before achieving stability, after which it maintains low conformational drift, indicative of high overall stability.

TEM was employed to analyze the morphology of Aβ42‐hIAPP co‐oligomers, AβO, scrambled Aβ, and hIAPP. The Aβ42‐hIAPP co‐oligomers exhibited an amorphous polymeric structure, featuring a central clustered core with radiating elongated fibers. In contrast, AβO appeared as spherical particles, SAβ formed larger non‐fibrous aggregates, and hIAPP displayed characteristic fibrous morphology (Figure 2H). Thioflavin‐T (ThT) analysis revealed that the control group maintained baseline fluorescence levels over 24 h with no protein aggregation. The Scrambled Aβ group exhibited a mild aggregation trend. The hIAPP group showed no change between 0–2.5 h, followed by a sharp increase thereafter. The AβO group showed a progressive increase in fluorescence, with an aggregation rate intermediate between Scrambled Aβ and hIAPP. Notably, the Aβ42‐hIAPP mixture group exhibited a rapid rise in fluorescence from the beginning, with the rate of increase slowing after 2 h but continuing to rise through to 24 h, demonstrating an aggregation pattern distinct from that of the individual proteins. In conclusion, hIAPP enhanced the aggregation rate of AβO to a certain extent, suggesting that hIAPP may interact with AβO, thereby altering its aggregation pathway (Figure 2I).

Real‐time cell analysis revealed that following treatment with 10 µm AβO or Aβ42‐hIAPP co‐oligomers, the AβO group exhibited cytotoxicity as early as 6 h post‐treatment (corresponding to the 24‐h time point), with a killing rate of approximately 12.88%. In contrast, the Aβ42‐hIAPP co‐oligomer group demonstrated significantly stronger toxicity, achieving a killing rate of 28.28%, which was approximately 2.19‐fold higher than that of the AβO group. A marked difference in the duration of toxicity was observed between the two treatments: By 96 h of culture, the cytotoxic effect of the Aβ42‐hIAPP co‐oligomer group had intensified to 6.26 times that of the AβO group (Figure 2J). Notably, dynamic monitoring data indicated that the cytotoxicity induced by Aβ42‐hIAPP co‐oligomers exhibited a sustained and progressively increasing trend. Conversely, cells in the AβO‐treated group recovered activity and resumed a normal proliferative status by 48 h post‐treatment. This suggests that AβO induces only transient early‐stage toxicity, where its initial, weaker damage is potentially reversed over time by cellular self‐repair mechanisms. In stark contrast, Aβ42‐hIAPP co‐oligomers elicit pronounced and persistent cytotoxic effects.

Unlike conventional induction methods [29], we adopted a stereotactic injection approach to deliver Aβ42‐hIAPP co‐oligomers, AβO, SAβ, or HEPES control solution precisely into the CO core using a micropump system (Figure 1A, green module). Subsequently, fluorescence imaging (792 nm) revealed that the fluorescently labelled injection solution had successfully reached the CO center and exhibited radial diffusion (Figure 2K). WB analysis of the Aβ protein demonstrated that both Aβ42‐hIAPP co‐oligomers and the AβO maintained stability before and after injection (Figure 2L). The Aβ42‐hIAPP co‐oligomers displayed greater heterogeneity in aggregate forms and higher molecular weights compared to AβO (Figure 2L). Following injection, COs were cultured until D190, then embedded in OCT‐embedded compound for histopathological analysis (Figure 1A, orange module) and culture supernatant metabolomic analysis (Figure 1A, blue module).

Data are represented as mean ± SD. *n* = 3 replicates per group (I).

### 2.3 Significant Dense Aβ Plaque Pathology in COs Induced by Aβ42‐hIAPP Co‐Oligomers

**FIGURE 3:**
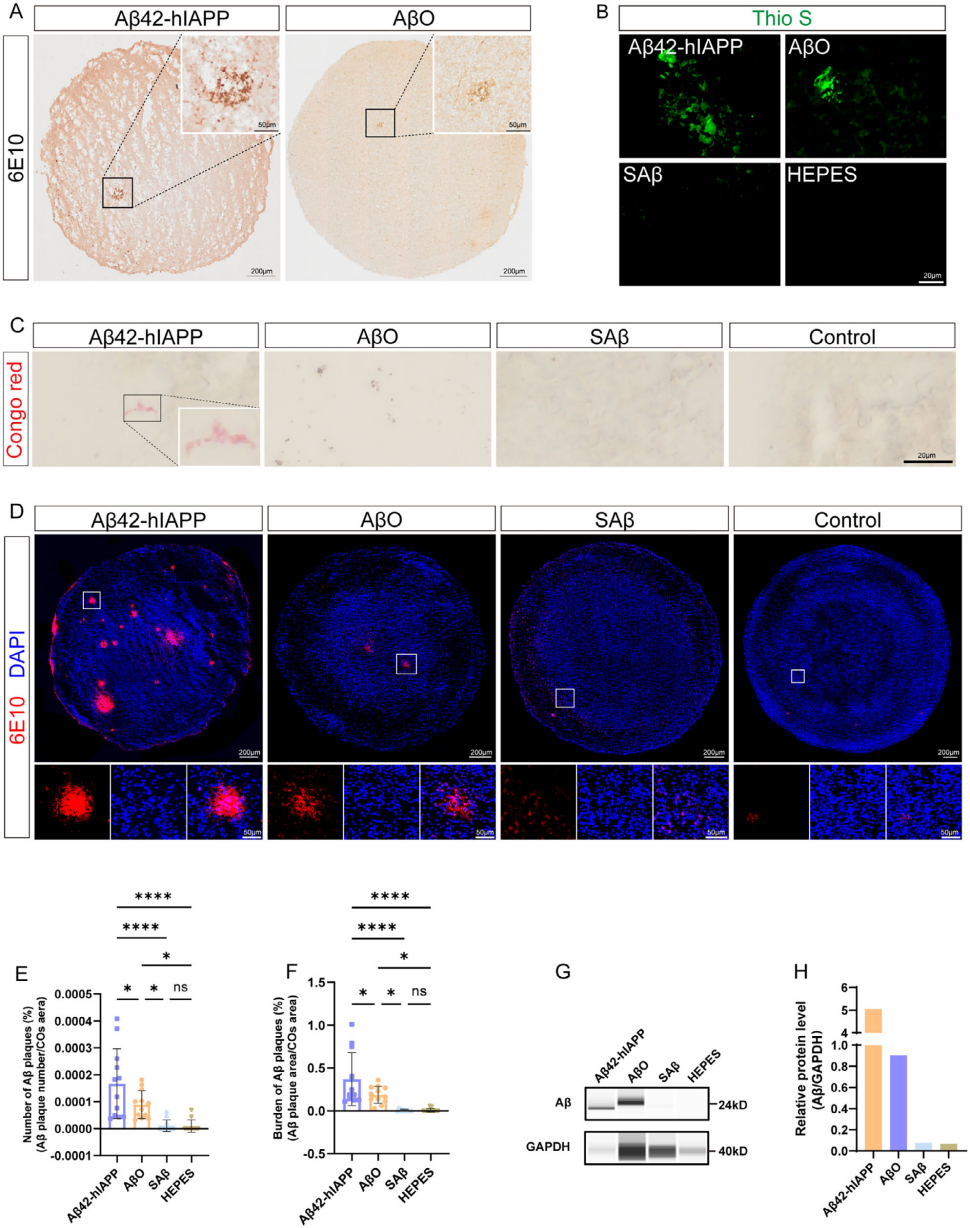
**Histologic staining of Aβ pathological features in COs induced by Aβ42‐hIAPP co‐oligomers** A. Immunohistochemical detection of Aβ plaques in COs using 6E10 antibody, comparing Aβ42‐hIAPP co‐oligomer and AβO groups. Scale bar: 200 µm, 50 µm. B. The detection of Aβ plaques by Thioflavin S staining. Scale bar: 20 µm. C. The detection of Aβ plaques in COs of Aβ42‐hIAPP co‐oligomers, AβO, SAβ, and HEPES group by Congo red staining; Scale bar: 20 µm. D‐F. Immunofluorescence staining (D) and quantitative analysis of the number (E) and burden (F) of Aβ plaques (6E10) in COs of Aβ42‐hIAPP co‐oligomers, AβO, SAβ, and HEPES groups. Scale bar: 200 µm and 50 µm. G‐H. Capillary‐based immunoassays of Aβ content (6E10 antibody) in COs of Aβ42‐hIAPP co‐oligomers, AβO, SAβ, and HEPES group (G), and normalized to GAPDH (H).

We assessed Aβ deposition in COs treated with Aβ42‐hIAPP co‐oligomers, AβO, SAβ, or HEPES buffer using 6E10 antibody staining. Both Aβ42‐hIAPP co‐oligomers and AβO induced dense Aβ plaques (Figure 3A, Figure S1E). Furthermore, Aβ plaques in these groups were detectable by standard methods used for identifying Aβ pathology in patient brain, including Thioflavin S (Figure 3B) and Congo Red staining (Figure 3C). Immunofluorescence staining results indicate that, compared to AβO‐treated COs, those exposed to Aβ42‐hIAPP co‐oligomers exhibited plaques with larger diameters and more defined outlines, resembling cored plaques with miliary foci. These features closely resembled the characteristic senile plaques observed in the cortex of AD patients. Aβ plaques induced by AβO were smaller [4]. A small number of granular deposits appeared intracellularly or extracellularly in SAβ and HEPES induced COs (Figure 3D). Quantitative analyzes demonstrated that the number and load of Aβ plaques were significantly increased in the Aβ42‐hIAPP co‐oligomer group, with a more modest increase observed in the AβO group (Figure 3E,F). Furthermore, the WES results corroborated the pathological staining findings, revealing higher Aβ protein levels in the Aβ42‐hIAPP co‐oligomer group than in the AβO group (Figure 3G,H). These findings indicate that Aβ42‐hIAPP co‐oligomers induce more severe Aβ‐like pathology in COs relative to AβO alone.

Data are represented as mean ± SD. *n* = 4 COs for each of the Aβ42‐hIAPP co‐oligomer, AβO, SAβ, and HEPES groups; 3 independent slices were analyzed per CO (E‐F). P‐values are calculated using One‐way ANOVA testing followed by a Tukey post‐hoc test. ns p>0.05, ^*^ *p* ≤ 0.05; ^****^ *p* ≤ 0.0001.

### 2.4 Tau Pathology Features of NFT with Ghost Tangle Morphology were Observed in COs Induced by Aβ42‐hIAPP Co‐Oligomers

**FIGURE 4:**
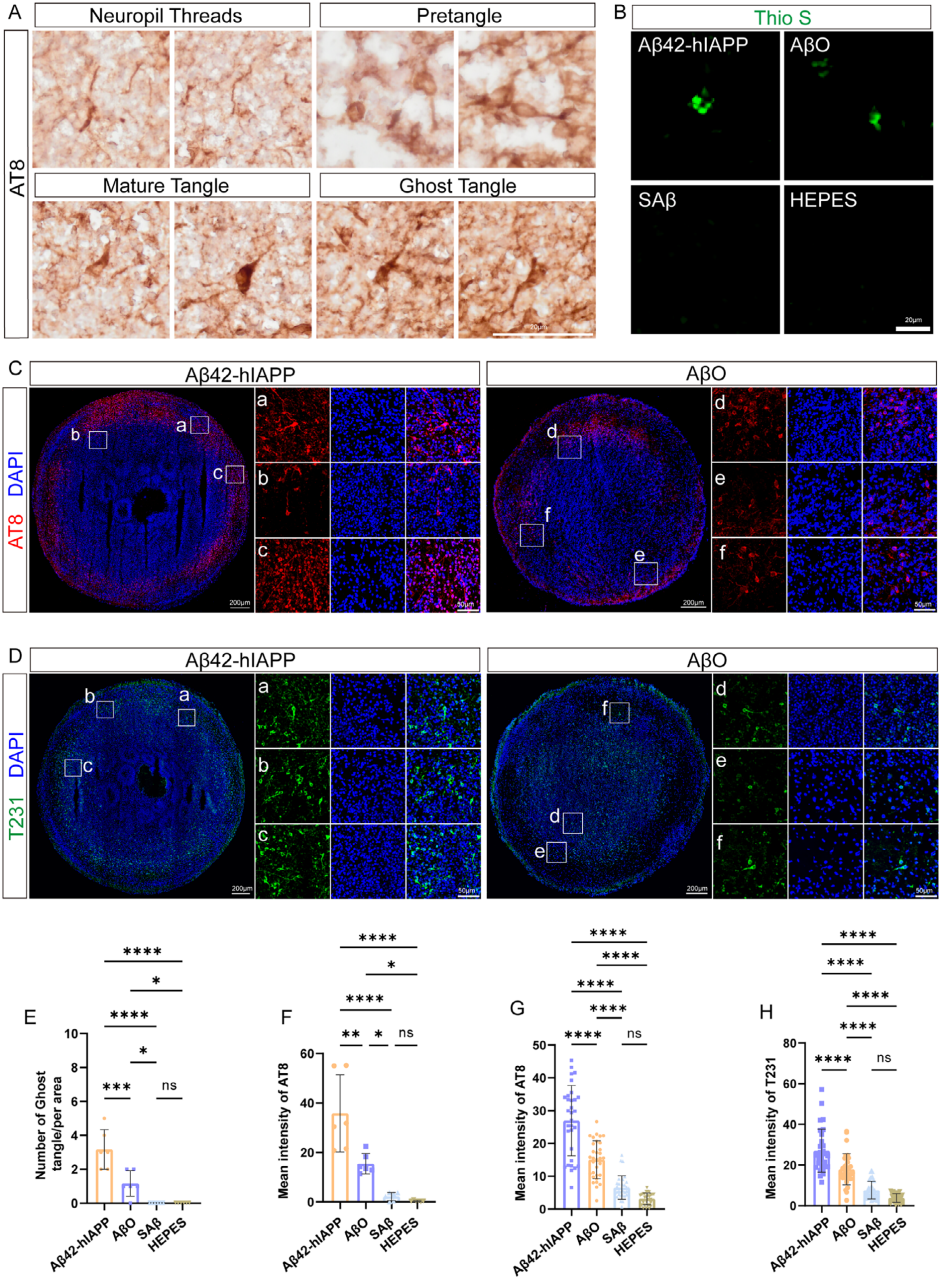
Histological staining of NFT pathological features in COs induced by Aβ42‐hIAPP co‐oligomers. A, E, F. Immunohistochemical staining analysis of NFT (AT8) at different maturity levels in COs treated with Aβ42‐hIAPP co‐oligomer, AβO, SAβ, and HEPES. Quantify the number of ghost tangles (E) and the level of p‐tau (F). Scale bar: 20 µm. B. The detection of mature NFTs by Thioflavin S staining. Scale bar: 20 µm. C‐D. Analysis of p‐tau levels in COs treated with Aβ42‐hIAPP co‐oligomers or AβO group by immunofluorescence staining with AT8 (C) and T231 (D) antibody. Scale bar: 200 µm, 50 µm. G‐H. Quantitative analysis of immunopositive results for AT8 (G) and T231 (H) in COs treated with Aβ42‐hIAPP co‐oligomers, AβO, SAβ, and HEPES groups.

The microtubule‐associated protein tau is aberrantly phosphorylated in AD brains and aggregates as paired helical filaments (PHFs) to form NFTs, another important pathological hallmark of AD. We used multiple site‐specific phosphorylated tau antibodies to assess NFT pathological changes. AT8 antibody staining revealed that COs from the Aβ42‐hIAPP co‐oligomer and AβO groups exhibited various tangle morphologies, including extracellular filamentous neuropil threads; perinuclear, diffusely stained ring‐ or droplet‐shaped pre‐NFTs; intraneuronal NFTs (iNFTs) containing aggregated filamentous cytoplasmic structures with flame‐like shapes; and extra‐neuronal NFTs (eNFTs) of protofibrils formed by neuronal nuclear atrophy and dislocation (ghost tangles) (Figure 4A and Figure S2A) [30]. Quantification showed Aβ42‐hIAPP co‐oligomer‐treated COs had significantly increased ghost tangle numbers and p‐tau levels compared to AβO‐treated COs (Figure 4E,F). The quantitative results of AT8 immunofluorescence staining in 6–8 layers of each CO further confirmed these findings (Figure 4C, G and Figure S2D). Thioflavin S staining further verified insoluble mature NFTs in Aβ42‐hIAPP co‐oligomer‐ and AβO‐treated COs (Figure 4B). Notably, AT8‐positive neuritic plaques were present in Aβ42‐hIAPP co‐oligomer‐ and AβO‐treated COs, with denser cores and more dispersed haloes in the Aβ42‐hIAPP co‐oligomer group (Figure S2F).

Extensive T231‐positive p‐tau signaling was observed in the Aβ42‐hIAPP co‐oligomer group, displaying flame‐like mature tangles and ring‐ or bead‐like pre‐NFT morphology (Figure 4D and Figure S2E). Quantitative results correlated with AT8 staining, showing the highest T231+ p‐tau expression in the Aβ42‐hIAPP co‐oligomer group, followed by the AβO group (Figure 4H). Additionally, sparse cells with diffuse granular T231+ p‐tau aggregates were also detected in the HEPES group, likely reflecting normal ageing during culture processes [31]. Capillary‐based immunoassays further verified elevated p‐tau levels in the Aβ42‐hIAPP co‐oligomer group (Figure S2B,C). Multiple pS396‐positive NFT types were identified in the Aβ42‐hIAPP co‐oligomer group, including pre‐NFTs (arrowheads) and mature NFTs (triangles), whereas only pre‐NFTs (arrowheads) were present in the AβO group (Figure S2G). Quantitative analysis demonstrated significantly higher pS396 expression in the Aβ42‐hIAPP co‐oligomer group compared to the AβO group (Figure S2H).

Notably, we observed a positive correlation between Aβ plaque formation and tau pathology (Table S1). The Aβ plaques burden correlated with the mean fluorescence intensity of AT8 (Pearson's *r* = 0.6423, *p* = 0.0179) and T231 (Pearson's *r* = 0.6797, *p* = 0.0106).

Data are represented as mean ± SD. *n* = 4 COs for each of the Aβ42‐hIAPP co‐oligomer, AβO, SAβ, and HEPES groups; *n* = 6 independent slices were analyzed for each group (E‐F); each CO analyzes 6–8 independent slices (G‐H). P‐values are calculated using One‐way ANOVA testing followed by a Tukey post‐hoc test. ns p>0.05, ^*^ *p* ≤ 0.05; ^**^ *p* ≤ 0.01; ^***^ *p* ≤ 0.001; ^****^ *p* ≤ 0.0001.

### 2.5 Significant Astrocyte Activation, Inflammatory Vesicle Formation, and Neuroinflammatory Pathology were Observed in COs Induced by Aβ42‐hIAPP Co‐Oligomers

**FIGURE 5:**
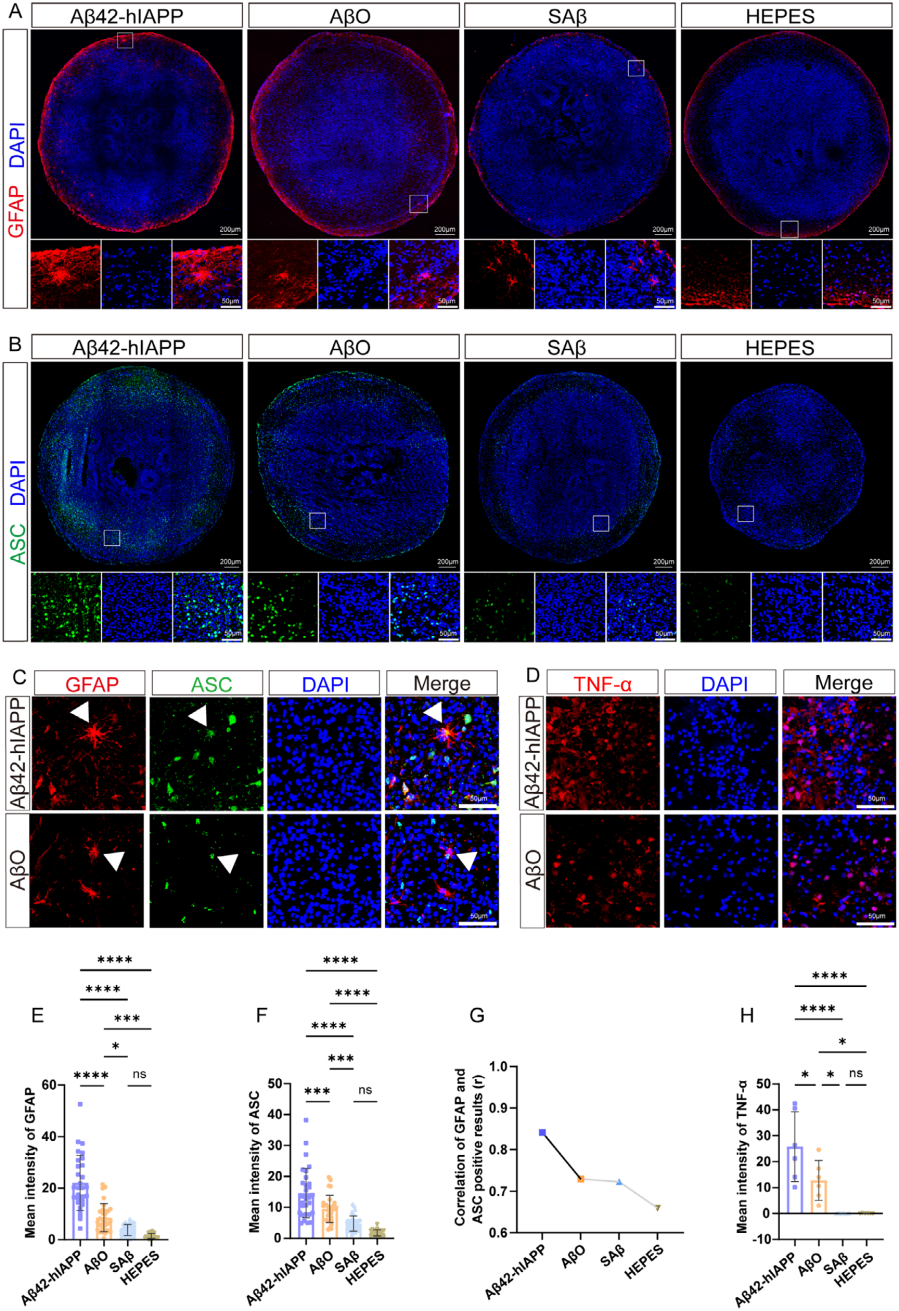
Histological staining of astrocytes, inflammatory vesicles, and inflammatory factors in COs induced by Aβ42‐hIAPP co‐oligomers. A, E. Immunofluorescence staining of astrocytes (A) and mean intensity quantification (E) in COs treated with Aβ42‐hIAPP co‐oligomers, AβO, SAβ, and HEPES. Scale bar: 200 µm, 50 µm. B, F. Immunofluorescence staining of ASC specks (A) and mean intensity quantification (E) in COs treated with Aβ42‐hIAPP co‐oligomers, AβO, SAβ, and HEPES. Scale bar: 200 µm, 50 µm. C. Merge image of astrocyte (GFAP) immunofluorescence staining (red) and ASC (green) in COs of Aβ42‐hIAPP co‐oligomer and AβO groups. Scale bar: 50 µm. D. Immunofluorescence staining of TNF‐α in COs treated with Aβ42‐hIAPP co‐oligomers or AβO. Scale bar: 50 µm. G. Correlation between mean GFAP and ASC immunofluorescence intensities in COs treated with Aβ42‐hIAPP co‐oligomers, AβO, SAβ and HEPES. H. TNF‐α expression levels in COs treated with Aβ42‐hIAPP co‐oligomers, AβO, SAβ and HEPES.

Neuroinflammation represents another key neuropathological feature of AD, primarily characterized by glial cell activation and inflammatory vesicle formation [32]. Our observations revealed astrocytes (GFAP‐positive) predominantly clustered on the CO surface. While astrocytes in the AβO group exhibited mild activation, those in the Aβ42‐hIAPP co‐oligomer group displayed significantly enlarged cytosol, more extensive branching, and greater morphological complexity. In contrast, the SAβ and HEPES groups showed resting astrocytes (Figure 5A). Quantitative analysis demonstrated the highest GFAP immunoreactivity in the Aβ42‐hIAPP co‐oligomer group, with intermediate levels in the AβO group (Figure 5E).

The inflammasome serves as the central signaling hub of inflammatory responses, with ASC speck formation being a hallmark of inflammasome activation [33, 34]. In this study, ASC‐positive specks in the Aβ42‐hIAPP co‐oligomer group were predominantly intracellular aggregates, though some showed extracellular diffusion, particularly at CO peripheries (Figure 5B). Quantitative analysis demonstrated significantly elevated ASC+ speck expression in the Aβ42‐hIAPP co‐oligomer group compared to the AβO group (Figure 5F). Notably, certain ASC+ specks appeared within and surrounding GFAP+ cells (Figure 5C, Figure S3A), exhibiting a strong positive correlation with GFAP immunoreactivity, most pronounced in the Aβ42‐hIAPP co‐oligomer group (Figure 5G and Table S3). These findings demonstrate that Aβ42‐hIAPP co‐oligomers induce more severe neuroinflammation in COs. Substantial tumor necrosis factor‐α (TNF‐α) was detected in both Aβ42‐hIAPP co‐oligomer and AβO groups (Figure 5D and Figure S3F), whereas the level of TNF‐α was significantly higher in the Aβ42‐hIAPP co‐oligomer group than in the AβO group (Figure 5H).

Notably, both Aβ plaque and tau pathology showed association with neuroinflammation (Tables S1,S2). The Aβ plaques burden correlated with GFAP (Pearson's *r* = 0.7506, *p* = 0.0031) and ASC (Pearson's *r* = 0.7952, *p* = 0.0012) mean fluorescence intensity. Similarly, T231 mean fluorescence intensity correlated with GFAP (Pearson's *r* = 0.9244, *p* < 0.0001) and ASC (Pearson's *r* = 0.8864, *p* < 0.0001) mean fluorescence intensities. Correspondingly, AT8 mean fluorescence intensity demonstrated correlation with GFAP (Pearson's *r* = 0.9181, *p* < 0.0001) and ASC (Pearson's *r* = 0.7841; *p* = 0.0003) mean fluorescence intensities.

Data are represented as mean ± SD. *n* = 4 COs for each of the Aβ42‐hIAPP co‐oligomer, AβO, SAβ and HEPES groups; each CO analyzes 6–8 independent slices (E‐F); 3 images (20×) are randomly selected for each CO (G); *n* = 6 independent slices were analyzed for each group (H). P‐values are calculated using One‐way ANOVA testing followed by a Tukey post‐hoc test. ns>0.05; ^*^ *p* ≤ 0.05; ^***^ *p* ≤ 0.001; ^****^ *p* ≤ 0.0001. The r value represents the Pearson correlation coefficient.

### 2.6 Aβ42‐hIAPP Co‐Oligomers Induced Significant Synaptic Damage in COs

**FIGURE 6:**
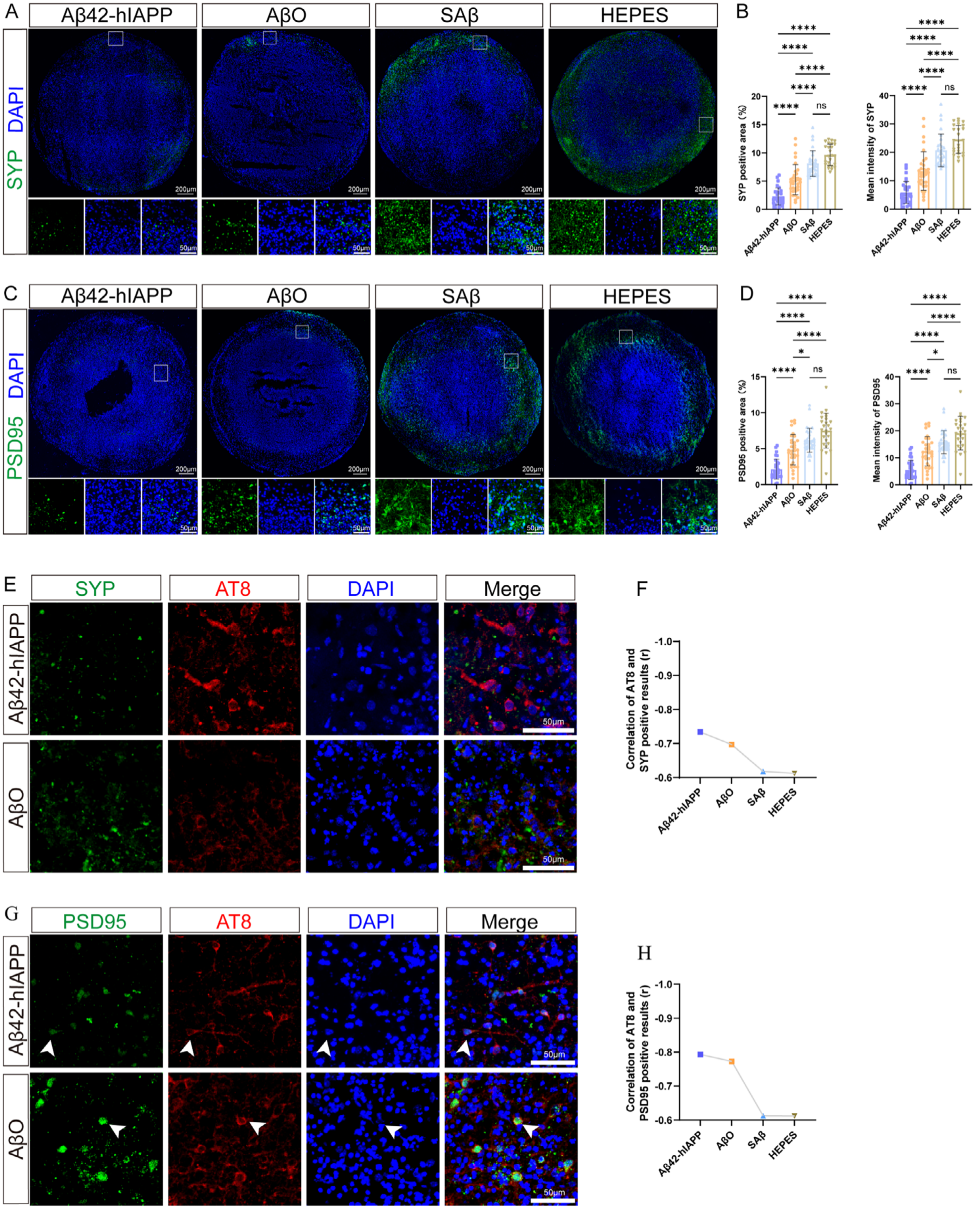
Histological staining of synaptic damage in COs induced by Aβ42‐hIAPP co‐oligomer. A. Images of SYP immunofluorescence staining in COs treated with Aβ42‐hIAPP co‐oligomers, AβO, SAβ, and HEPES. Scale bar: 200 µm, 50 µm. B. Quantification of SYP in COs of the Aβ42‐hIAPP co‐oligomer, AβO, SAβ, and HEPES groups. C. Images of PSD95 immunofluorescence staining in COs treated with Aβ42‐hIAPP co‐oligomers, AβO, SAβ, and HEPES. Scale bar: 200 µm, 50 µm. D. Quantification of PSD95 in COs of the Aβ42‐hIAPP co‐oligomer, AβO, SAβ, and HEPES groups. E. Merge image of p‐tau (AT8) immunofluorescence staining (red) and SYP (green) in COs of Aβ42‐hIAPP co‐oligomer and AβO groups. Scale bar: 50 µm. F. Correlation between mean immunofluorescence intensities of AT8 and SYP co‐staining in COs treated with Aβ42‐hIAPP co‐oligomers, AβO, SAβ, and HEPES. G. Merge image of p‐tau (AT8) immunofluorescence staining (red) and PSD95 (green) in COs of Aβ42‐hIAPP co‐oligomer and AβO groups. Scale bar: 50 µm. H. Correlation between mean immunofluorescence intensities of AT8 and PSD95 co‐staining in COs treated with Aβ42‐hIAPP co‐oligomers, AβO, SAβ, and HEPES.

Synaptic structural and functional abnormalities are key contributors to AD pathogenesis [35]. In this study, synaptic damage in COs was assessed using antibodies targeting the presynaptic marker synaptophysin (SYP) and the postsynaptic marker postsynaptic density‐95 (PSD95). Quantitative analysis of 6–8 layers in each CO revealed that the SYP‐positive density and mean fluorescence intensity were significantly reduced in the Aβ42‐hIAPP co‐oligomer group compared to all other groups, with the AβO group exhibiting the next most pronounced reduction (Figure 6A,B). Immuno‐co‐staining of SYP with AT8 demonstrated a significant negative correlation between their mean fluorescence intensities (Figure 6E,F; Figure S3B and Table S3). PSD95 displayed a similar pathological pattern to SYP. The Aβ42‐hIAPP co‐oligomer group exhibited marked morphological disruption of PSD95 alongside significantly diminished immunopositivity (Figure 6C,D). Immunolocalization analysis confirmed co‐localization of AT8 and PSD95 (Figure 6G and Figure S3C), with a significant negative correlation between their signals, most pronounced in the Aβ42‐hIAPP co‐oligomer group (Figure 6H and Table S3). Collectively, these findings indicate that Aβ42‐hIAPP co‐oligomers induce more severe synaptic damage in COs than AβO alone.

Further analysis demonstrated associations between both Aβ plaques and tau pathology with synaptic damage (Table S1,S2). Aβ plaques burden showed significant negative correlations with mean fluorescence intensity of SYP (Pearson's *r* = −0.6643, *p* = 0.0133) and PSD95 (Pearson's *r* = −0.6617, *p* = 0.0138). Similarly, T231 intensity correlated negatively SYP (Pearson's *r* = −0.7654, *p* = 0.0005) and PSD95 (Pearson's *r* = −0.8189, *p* = 0.0001) intensity. The strongest correlations were observed between AT8 intensity and synaptic markers: SYP (Pearson's *r* = −0.8893, *p* < 0.0001) and PSD95 (Pearson's *r* = −0.9368, *p* < 0.0001).

Data are represented as mean ± SD. *n* = 4 COs for each of the Aβ42‐hIAPP co‐oligomer, AβO, SAβ, and HEPES groups, each CO analyzes 6–8 independent slices (B, D); 3 images (20×) are randomly selected for each CO (F, H). P‐values are calculated using One‐way ANOVA testing followed by a Tukey post‐hoc test. ns>0.05; ^*^ *p* ≤ 0.05; ^****^ *p* ≤ 0.0001. The r value represents the Pearson correlation coefficient.

### 2.7 Aβ42‐hIAPP Co‐Oligomers Induced Significant Neuronal Death and Loss in COs

**FIGURE 7:**
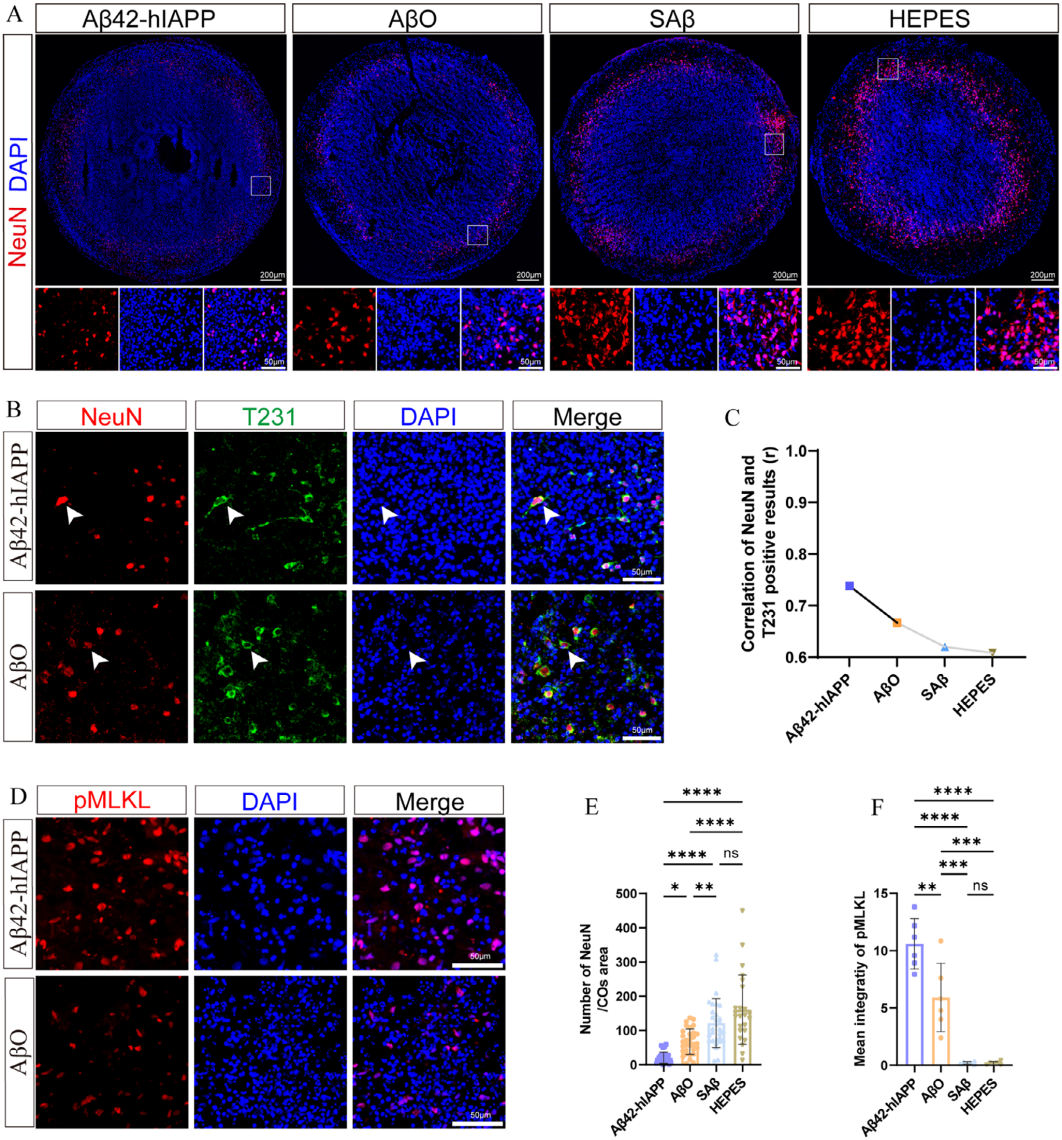
Histological staining of neurodegeneration in COs induced by Aβ42‐hIAPP co‐oligomers. A. Images of NeuN immunofluorescence staining in COs treated with Aβ42‐hIAPP co‐oligomers, AβO, SAβ, and HEPES. Scale bar: 200 µm, 50 µm. B. Merge image of p‐tau (T231) immunofluorescence staining (green) and NeuN (red) in COs of Aβ42‐hIAPP co‐oligomer and AβO groups. Scale bar: 50 µm. C. Correlation between mean fluorescence intensities of T231 and NeuN in COs treated with Aβ42‐hIAPP co‐oligomers, AβO, SAβ, and HEPES. D. Immunofluorescence staining images of pMLKL in COs treated with Aβ42‐hIAPP co‐oligomer and AβO group. Scale bar: 50 µm. E. The density quantification of NeuN in COs of the Aβ42‐hIAPP co‐oligomer, AβO, SAβ, and HEPES groups. F. Quantification of pMLKL mean intensity in COs of the Aβ42‐hIAPP co‐oligomers, AβO, SAβ, and HEPES groups.

Extensive neuronal degeneration represents a key pathological hallmark of cognitive impairment in AD patients. While we observed moderate neuronal atrophy in AβO‐treated COs compared to SAβ and HEPES control groups, the morphological atrophy of NeuN+ neurons was most severe in the Aβ42‐hIAPP co‐oligomer group (Figure 7A). Quantitative analysis revealed significantly reduced NeuN+ neuronal density in the Aβ42‐hIAPP co‐oligomer group, with a 3.2‐fold increase in neuronal death relative to AβO treatment (Figure 7E). Furthermore, we detected T231+ p‐tau tangles surrounding NeuN+ neurons in Aβ42‐hIAPP co‐oligomer‐treated COs. Neurons containing p‐tau tangles exhibited varying degrees of nuclear atrophy (Figure 7B and Figure S3D). Notably, we identified a significant negative correlation between the NeuN density and T231 levels (Figure 7C and Tables S3). These findings demonstrate that Aβ42‐hIAPP co‐oligomers cause substantially greater neuronal damage and loss in COs compared to AβO alone. Notably, Aβ plaques and p‐tau showed significant associations with neuronal loss (Tables S2,S3). Aβ plaques burden demonstrated a negative correlation with NeuN neuronal density (Pearson's *r* = −0.6467, *p* = 0.0169). Furthermore, NeuN density correlated negatively correlated with both T231 (Pearson's *r* = −0.8518, *p* < 0.0001) and AT8 fluorescence intensity (Pearson's *r* = −0.8570, *p* < 0.0001).

Moreover, pMLKL expression, a marker of necrotic cell death, was detected in perinuclear or intranuclear regions of neuronal cells in Aβ42‐hIAPP co‐oligomer and AβO groups (Figure 7D and Figure S3E). Notably, pMLKL levels were highest in the Aβ42‐hIAPP co‐oligomer group, with the AβO group exhibiting intermediate expression (Figure 7F).

Data are represented as mean ± SD. *n* = 4 COs for each of the Aβ42‐hIAPP co‐oligomer, AβO, SAβ, and HEPES groups; 3 images (20×) are randomly selected for each CO (C); each CO analyzes 6–8 independent slices (E); *n* = 6 independent slices were analyzed for each group (F). P‐values are calculated using One‐way ANOVA testing followed by a Tukey post‐hoc test. ns>0.05; ^*^ *p* ≤ 0.05; ^**^ *p* ≤ 0.01; ^***^ *p* ≤ 0.001; ^****^ *p* ≤ 0.0001. The r value represents the Pearson correlation coefficient.

### 2.8 Dynamics of Metabolomics in CO Cultures Induced by Aβ42‐hIAPP Co‐Oligomers

We analyzed the dynamic metabolomics profiles of CO cultures treated with Aβ42‐hIAPP co‐oligomers, AβO, SAβ, or HEPES at multiple time points: pre‐injection (D0) and post‐injection (D15, D45, and D90) (Figure 1A, blue module).

**FIGURE 8:**
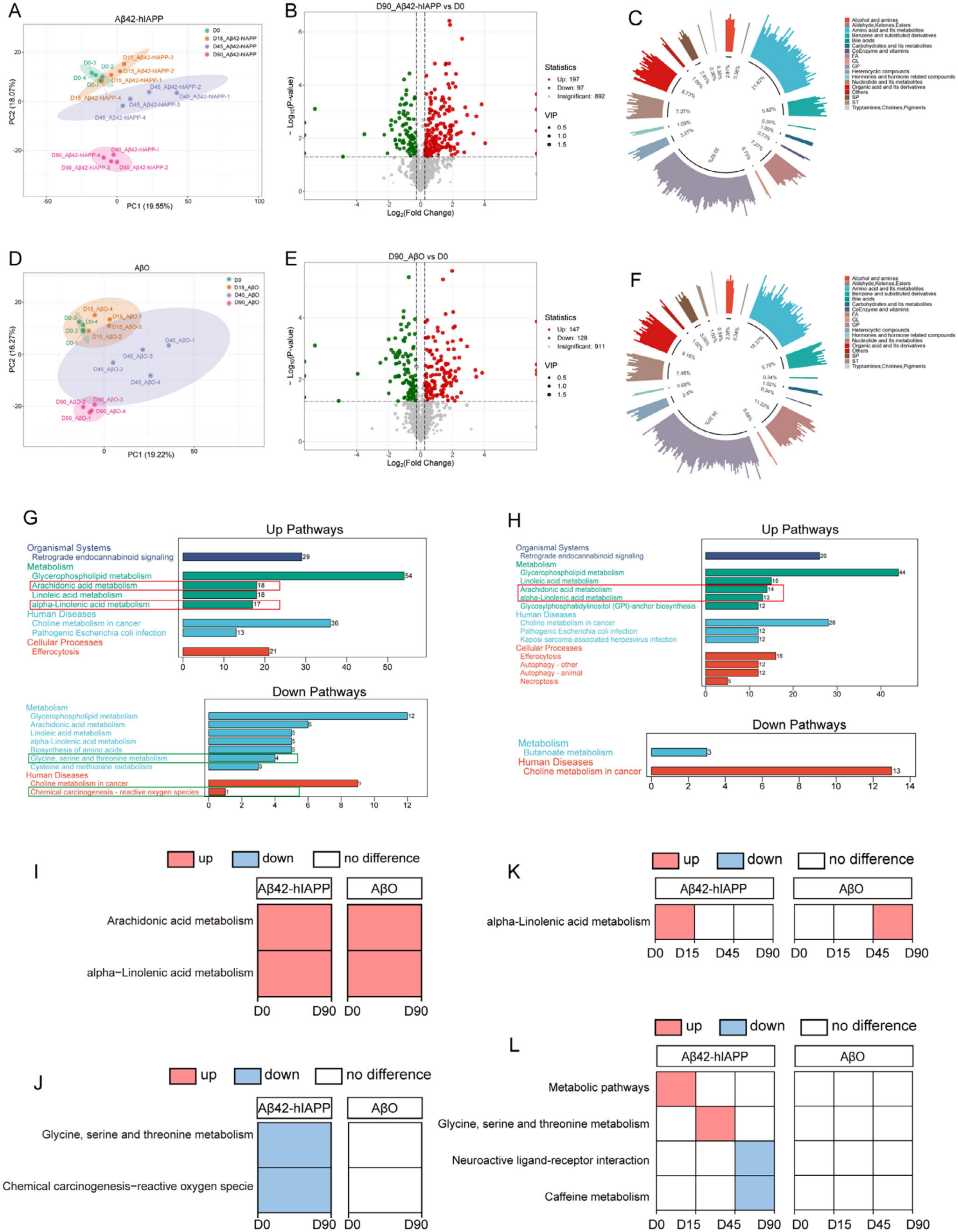
Metabolomic analysis for COs cultures of Aβ42‐hIAPP co‐oligomer and AβO groups during D0‐D90. A, D. Principal component analysis (PCA) of Aβ42‐hIAPP co‐oligomer (A) and AβO group (D) at D0, D15, D45, and D90. Points marked with the same color represent four replicates of each group. B, E. Volcano plots showing DEM during D0‐D90 in Aβ42‐hIAPP co‐oligomer (B) and AβO group (E). Screening of DEM required both Foldchange (FC) > 1.2, VIP > 1, and *P*‐value < 0.05 (Student's test). Green spots indicate down‐regulated DEM; red spots indicate up‐regulated DEM; and grey spots indicate metabolites with insignificant differences detected. X axis: FC; y axis: log10 (P‐values). C, F. Plots of occupied circles for the first‐level classification of Aβ42‐hIAPP co‐oligomer (C) and AβO group (F), showing the categorization information of the differential metabolites. The colors of the outer circles and the length of the columns represent the different classes of substances and their relative content levels. The inner circle is the ratio of the amount of each class of substance to the amount of all substances. G‐H. Significantly up‐ and down‐regulated KEGG pathways enriched in Aβ42‐hIAPP co‐oligomer (G) and AβO group (H) during D0‐D90. Y‐axis: Significantly enriched pathways across different KEGG Level 1 classifications. X‐axis: Number of DEMs enriched into each pathway. I‐J. In systematic metabolomics (D0‐D90), KEGG pathways common to Aβ42‐hIAPP co‐oligomer and AβO group (I). Unique KEGG pathways of Aβ42‐hIAPP co‐oligomer group (J). K‐L. In dynamic metabolomics (D0‐D15; D15‐D45; D45‐D90), KEGG pathways common to Aβ42‐hIAPP co‐oligomer and AβO group (K). Unique KEGG pathways of Aβ42‐hIAPP co‐oligomer group (L). Red and blue squares indicate KEGG pathways that were up‐regulated and down‐regulated, respectively, during this time period, and white squares indicate no change.

Firstly, principal component analysis (PCA) plots of the Aβ42‐hIAPP co‐oligomer, AβO, SAβ, and HEPES groups at D0, D15, D45, and D90 indicate that their metabolites progressively dissociate with increasing induction duration (Figure 8A,D and Figure S4A,D). Subsequently, we analyzed the systemic metabolomics (D0–D90). Volcano plots identified 294, 275, 264, and 279 differentially expressed metabolites (DEM) in the Aβ42‐hIAPP co‐oligomer, AβO, SAβ, and HEPES groups, respectively (Figure 8B,E and Figure S4B, E). The predominant metabolite class in all groups was glycerophospholipids (GP) (Figure 8C,F and Figure S4C,F). Functional enrichment analysis of these metabolites showed that up‐regulated and down‐regulated KEGG pathways in the Aβ42‐hIAPP co‐oligomer, AβO, SAβ, and HEPES groups, respectively (Figure 8G,H and Figure S4G,H). Subsequently, we analyzed dynamic metabolomic changes in the Aβ42‐hIAPP co‐oligomer, AβO, SAβ, and HEPES groups across different culture stages (D0‐D15, D15‐D45, D45‐D90), identifying DEM (Figure S4I–L) and their associated metabolic pathways (Table S4).

Subsequently, dynamic metabolomics (D0‐D15, D15‐D45, D45‐D90) was integrated with systematic metabolomics (D0‐D90) for the Aβ42‐hIAPP co‐oligomer, AβO, SAβ, and HEPES groups. First, we identified common differential metabolic pathways between the Aβ42‐hIAPP co‐oligomer and AβO groups, when compared against the SAβ and HEPES groups (Figure 8G,H, red boxes). Notably, alpha‐Linolenic acid metabolism was altered during early induction (D0‐D15) in the Aβ42‐hIAPP co‐oligomer group but during late induction (D45‐D90) in the AβO group (Figure 8I,J), with phosphatidylcholine (PC) identified as a common DEM (Table S5). Second, we examined metabolic pathways unique to the Aβ42‐hIAPP co‐oligomer group by comparison with the AβO, SAβ, and HEPES groups. Systematic metabolomic (D0‐D90) revealed that the Aβ42‐hIAPP co‐oligomer group displayed unique metabolism pathways (Figure 8G, green box). These pathways contained distinct metabolite profiles: chemical carcinogenesis‐reactive oxygen species featured amino acid derivatives, while glycine, serine, and threonine metabolism included phosphatidylserine (PS), choline, and amino acids (Table S6). Furthermore, four distinct differential metabolic pathways were identified in the dynamic metabolomic profile of Aβ42‐hIAPP co‐oligomers (Figure 8L). During early induction (D0‐D15), metabolic pathway alterations involved PC, phosphatidylethanolamine (PE), and plasmalogen PE (PE‐P) (Table S6). The mid‐induction phase (D15‐D45) showed modifications in glycine, serine, and threonine metabolism, characterized by changes in PS and amino acids. Late induction (D45‐D90) demonstrated alterations in both neuroactive ligand‐receptor interaction and caffeine metabolism. The neuroactive ligand‐receptor interaction pathway exhibited changes in sulfonic acids, hormones, and hormone‐related compounds, amines, and lysophosphatidic acid (LPA). Conversely, caffeine metabolism was associated with variations in nucleotides and their metabolites (Table S6).

To further validate whether the differential metabolite PC can enhance cell membrane integrity, we conducted a PC supplementation experiment using SH‐SY5Y cells. The results revealed that compared to the AβO group and the Aβ42‐hIAPP co‐oligomer group without PC, the cell viability rates in the AβO+PC group and the Aβ42‐hIAPP+PC group showed a significant increase. Pure PC had no significant effect on cell viability rates. This indicates that PC protects cells from the toxicity of Aβ42‐hIAPP co‐polymers and AβO (Figure S5A). Subsequently, we performed targeted validation of the identified differential metabolite LPA using EDG2 (also known as LPA1), a G protein‐coupled receptor for LPA. The results revealed a significant increase in EDG2 levels in COs induced by Aβ42‐hIAPP co‐oligomers. This indicates that Aβ42‐hIAPP co‐oligomers promote EDG2 receptor activation via LPA (Figure S5B).

*N* = 4 biologically independent samples for each of the Aβ42‐hIAPP co‐oligomer, AβO, SAβ, and HEPES groups.

## 3 Discussion

In this study, we repeatedly administered either Aβ42‐hIAPP co‐oligomers or AβO, to hiPSC‐derived CO centers and characterized the AD pathological using histopathological methods. The results demonstrated that Aβ42‐hIAPP co‐oligomer‐induced COs displayed pathological features more closely resembling those of advanced AD as compared to AβO alone. These included denser Aβ plaques with larger diameters and increased abundance, a higher prevalence of mature tangles and ghost‐tangle NFT morphology, exacerbated neuroinflammation and synaptic damage, and neuronal death levels 3.2 times greater than those in the AβO group. These findings suggest that T2DM not only significantly enhances the neurotoxicity but also drives hiPSC‐derived COs to recapitulate hallmark AD pathological features. Dynamic metabolomic analysis of CO cultures revealed that Aβ42‐hIAPP co‐oligomers upregulated the same two differential metabolic pathways as AβO, alpha‐linolenic acid metabolism and arachidonic acid metabolism, from D0‐D90. The enrichment of these pathways correlated with elevated levels of the DEM PC, which may be associated with Aβ triggering the pathological cascade in AD. Further analysis identified five distinct differential metabolic pathways in Aβ42‐hIAPP co‐oligomer group. The metabolic pathways showed upregulation during D0‐D15, with PC and PE as the principal upregulated metabolites. Similarly, neuroactive ligand‐receptor interaction exhibited upregulated from D45‐D90, accompanied by increased levels of LPA. Conversely, glycine, serine, and threonine metabolism demonstrated downregulation at both D15‐D45 and D0‐D90 intervals, with significantly reduced PS levels. Caffeine metabolism was downregulated throughout D0‐D90, showing decreased enrichment of nucleotides and their metabolites. Similarly, the chemical carcinogenesis‐reactive oxygen species pathway displayed downregulation during D0‐D90, with reduced levels of enriched amino acid derivatives. These specific alterations are likely to be correlated to the primary mechanism through which Aβ42‐hIAPP co‐oligomer exacerbates neurotoxicity and accelerates AD pathology.

The classical Aβ cascade hypothesis suggests that Aβ deposition initiates AD pathogenesis [36], leading to NFT and subsequent AD pathological changes [37]. Previous studies have demonstrated that AβO induces Aβ deposition and tau pathology in COs^17^ and neurons [26]. In this study, Aβ42‐hIAPP co‐oligomer‐induced COs exhibited larger, more numerous dense Aβ plaques that more closely resembled those in the brains of AD patients [4]. Meanwhile, the p‐tau expression level was significantly higher in the Aβ42‐hIAPP co‐oligomer group than in the AβO group. Furthermore, the co‐oligomer group exhibited more ghost tangles, resembling those found in the brains of patients with advanced AD, whereas the AβO group displayed a greater variety of pre‐ and mature tangles [30, 38]. These findings were corroborated by thioflavin‐S‐positive staining. This suggests that the AβO group induced Aβ and tau pathology characteristic of early‐stage AD, whereas T2DM‐driven Aβ (via Aβ42‐hIAPP co‐oligomers) promoted features resembling more advanced AD pathology. Notably, neuritic plaques emerged in COs treated with Aβ42‐hIAPP co‐oligomers. Given that Aβ plaques may exacerbate tau pathology by facilitating neuritic plaque aggregation [39], these plaques could represent a critical link between Aβ and tau pathology [5].

In addition to Aβ plaques and tau pathology, COs treated with Aβ42‐hIAPP co‐oligomers showed activated astrocytes with more hypertrophied cytosol and complex morphology compared to those treated with AβO alone. These astrocytes more closely resembled those found in the brains of AD patients [40]. We also observed significantly higher levels of ASC speck and TNF‐α in the Aβ42‐hIAPP co‐oligomer group than in the AβO group. Previous studies reported that activated astrocytes and elevated TNF‐α were detected in the brain of T2DM monkeys [41], further supporting that T2DM exacerbates Aβ‐induced neuroinflammation. A previous study found that synaptic damage was induced by the coculture of AD patient brain extracts with COs [42]. In this study, the AβO group showed the same synaptic damage in COs. However, the degree of synaptic damage in COs induced by the Aβ42‐hIAPP co‐oligomers was significantly greater than that in the AβO group, consistent with the observation that synaptic damage is more severe in advanced AD patients than that in early‐stage AD patients [43]. Moreover, we found that expression of the necroptosis marker pMLKL was significantly higher in the Aβ42‐hIAPP co‐oligomer group than in the AβO group, similar to pMLKL levels detected in the brains of AD patients [44]. Importantly, matching the extensive neuronal loss seen in later‐stage AD patients [45], we observed significantly more neuronal damage and death in the Aβ42‐hIAPP co‐oligomer group, approximately 3.2‐fold higher than in the AβO group. This result was also confirmed by previous cellular studies [26]. These findings demonstrate that T2DM markedly enhances Aβ neurotoxicity, resulting in more severe synaptic damage, increased neuronal deaths, and features of more advanced AD pathology.

Notably, the total protein content injected into the COs was across all groups in this study. While the Aβ42‐hIAPP co‐oligomer group contained both Aβ42 and hIAPP proteins, the AβO group contained only the Aβ42 protein. Consequently, the Aβ42 concentration in the AβO group was 2‐fold higher than in the Aβ42‐hIAPP co‐oligomer group. Despite this lower Aβ42 concentration, Aβ42‐hIAPP co‐oligomers induced approximately 3.2‐fold more neuronal death and more severe AD‐like pathology in COs compared to AβO alone. These findings demonstrate that Aβ42‐hIAPP co‐oligomers, formed by the interaction of Aβ1‐42 and hIAPP, exhibit significantly enhanced neurotoxicity and better recapitulate the characteristic pathology observed in AD patients.

PC plays a crucial role in neuronal cell membrane synthesis and maintenance of synaptic function. In vitro studies have demonstrated that elevated PC levels protect neurons against Aβ1‐42‐induced damage [46]. Clinical studies have further identified a positive correlation between PC levels and Aβ deposition in serum metabolites of AD patients [47]. In our study, the Aβ42‐hIAPP co‐oligomer and AβO groups exhibited increased PC content in the alpha‐linolenic acid metabolism and arachidonic acid metabolism pathways. This suggests that PC available for neuronal membrane synthesis is depleted, compromising the structural integrity of the neuronal bilayer membrane and synaptic function, ultimately resulting in reduced neuroplasticity [48]. Aβ has been found to interact with neuronal membranes and membrane receptors, exacerbating membrane damage and cell apoptosis [49, 50]. Therefore, we hypothesize that the injected Aβ may have impaired the integrity of the neuronal membrane and disrupted synaptic function, thereby diminishing neural plasticity. Under these conditions, intracellular Aβ is released into the extracellular space, where it accumulates excessively to form senile plaques while triggering the Aβ cascade response. Notably, the Aβ42‐hIAPP co‐oligomer group has larger aggregates, which may accelerate membrane structural disruption and advance the pathological progression of AD. This hypothesis was further validated in our supplementary experiments, demonstrating that exogenous PC provides significant protection against cell damage induced by Aβ42‐hIAPP and AβO.

For the Aβ42‐hIAPP co‐oligomer group, multiple metabolites, including PC, PE, and PE‐P, exhibited changes in the pre‐induction period of the Metabolic pathways. Elevated levels of PC in the culture medium indicated that Aβ42‐hIAPP co‐oligomer had damaged the cell and mitochondrial membrane structure. PE primarily contributes to maintaining mitochondrial membrane structure and function [51]. PE has been found converted into PC within the brain via methylation pathways [52]. At this point, the upregulation of PE may represent a compensatory transformation occurring to synthesize PC for the maintenance of cell membrane integrity. Concurrently, increased PE‐P levels helped preserve membrane integrity by participating in lipid raft domain formation [53]. However, this metabolic compensation mechanism is insufficient to maintain membrane homeostasis. Thus, during early induction, Aβ42‐hIAPP co‐oligomers may have induced metabolic imbalance in COs, laying the groundwork for AD pathology progression.

With prolonged induction time, the Aβ42‐hIAPP co‐oligomer group exhibited a progressive decline in PS levels within the glycine, serine, and threonine metabolism pathway. PS can be synthesized from PC via enzymatic hydrolysis [54]. PS protects neurons from Aβ toxicity by inhibiting mitochondria‐dependent apoptotic pathways [55] and may contribute to diabetes development by targeting mitochondria to influence insulin secretion and signalling [56]. Moreover, reduced PS levels have been found in the brains of AD patients and diabetic rat models [57, 58]. The reduction in PS levels in the Aβ42‐hIAPP co‐oligomer group may be associated with persistent mitochondrial membrane damage leading to dysfunction, which promotes the development of AD pathology. During the later stages of induction, Aβ42‐hIAPP co‐oligomers increased levels of LPA, taurine, and palmitoylethanolamide (PEA) in the neuroactive ligand‐receptor interaction pathway within COs. Elevated LPA levels were found to promote AD pathology, including Aβ deposition, p‐tau aggregation, and neuronal death, through multiple mechanisms [59, 60, 61]. Our targeted validation in the Aβ42‐hIAPP co‐oligomer group also confirmed that elevated LAP activated increased EDG2 receptor levels. Furthermore, LAP promotes TNF‐α production [62], and elevated TNF‐α levels were similarly observed in our Aβ42‐hIAPP co‐oligomer group. At this point, elevated levels of PEA and taurine may exert neuroprotective and anti‐inflammatory effects [63, 64]. Therefore, we hypothesize that Aβ42‐hIAPP co‐oligomer modulates inter‐neuronal communication and neuroinflammation via the neuroactive ligand‐receptor interaction pathway, simultaneously promoting AD pathogenesis and neuronal death while exerting compensatory anti‐inflammatory.

Meanwhile, nucleotide and its metabolites in the caffeine metabolism pathway of Aβ42‐hIAPP co‐oligomer group, including xanthosine, xanthine, and theobromine, showed decreased levels during late induction. These compounds are all associated with caffeine synthesis and metabolism. Caffeine demonstrates neuroprotective effects in AD, slowing the disease progression through antioxidant activity and reduced Aβ deposition [65]. Therefore, we speculate that the downregulation of the caffeine metabolic pathway may accelerate the diminished cellular antioxidant capacity and impaired Aβ clearance, while simultaneously weakening neuroprotective mechanisms. Furthermore, Aβ42‐hIAPP co‐oligomers reduce levels of amino acid derivatives in the chemical carcinogenesis‐reactive oxygen species pathway, including S‐(5‐Adenosy)‐L‐homocysteine (SAH), a methyltransferase‐catalyzed product of S‐ adenosylmethionine (SAM). The observed SAH may reflect the mitochondrial dysfunction occurring during late induction stages, subsequently impairing SAM synthesis [66, 67]. These findings require further targeted studies to establish a causal relationship between these metabolic alterations and the pathogenesis of AD.

In this study, direct injection of AβO into the center of COs induced neuronal death in our study, whereas coculture with AβO [17] or serum [18] did not induce this phenotype. This is likely due to the failure of the induction solution to penetrate the organoid interior, thereby preventing the formation of AD pathological features. Thus, microinjection techniques hold greater promise than coculture methods for inducing more advanced AD pathology. In this study, Aβ42‐hIAPP co‐oligomer and AβO were intermittently and repeatedly microinjected into the CO center to simulate chronic exposure to pathological conditions within the brains of AD patients. After a 90‐day observation period, AD pathology was assessed using histopathological methods. Interestingly, the AD pathology in COs was primarily localized to the interior near the margins, overlapping with the distribution of neuronal (MAP2, GFAP, NeuN) and ventricular (SOX2, TUJ1, SATB2, CTIP2, TBR2) markers. This may occur because the outer layer of the CO efficiently absorbs nutrients from the medium, leading to higher neuronal activity [68]. Alternatively, the peptide solution injected into the CO diffuses outward from the center, which could prolong the presence of Aβ42‐hIAPP co‐oligomers or AβO in the outer tissue layer.

Although our study developed a sAD CO model that recapitulates the more advanced pathological features of AD using Aβ42‐hIAPP co‐oligomers, several limitations remain. The CO system in our study lacked a vascular system. This absence not only restricted nutrient delivery to the CO centers, limiting the duration of long‐period experiments, but also failed to simulate interactions with the blood‐brain barrier. Concurrently, the absence of microglia in COs limits the contribution of neuroinflammation to the pathophysiological processes of AD. Notably, this microglia deficiency might explain why AβO induces neuronal death in COs and glutamatergic neurons [26], but not in cynomolgus monkey brains [13]. New techniques have been developed to create COs containing vascular systems and microglia. In subsequent studies, we plan to integrate these two components, employ multiple iPSC cell lines, and expand sample sizes to construct more refined sAD models. Although we adhered to strict operating procedures, used identical peptide concentrations, and confirmed no leakage via fluorescence imaging, we could not eliminate the mechanical damage arising from the injection procedure itself. Moreover, organoid culture fluids struggle to replicate the complexity of the in vivo fluid environment. Although we have identified metabolites associated with AD pathology in organoid culture fluid, we shall validate through functional experiments how these selected metabolites contribute to the pathogenesis of AD. Finally, although we have constructed organoid models that can simulate pathology more resembling advanced AD, the evaluation of cognitive and memory impairment cannot be achieved within the organoid system. Therefore, we propose to use Aβ42‐hIAPP co‐oligomers to develop NHP models displaying clinical AD phenotypes in future studies. Additionally, we will systematically investigate the molecular mechanism by which T2DM accelerates the AD pathological cascade, employing a multi‐omics approach encompassing behavioral phenotyping, histopathology, transcriptomics, proteomics, and metabolomics.

In conclusion, this study modelled T2DM using hIAPP and employed Aβ42‐hIAPP co‐oligomers, formed by the interaction of Aβ1‐42 and hIAPP, to induce significantly stronger neurotoxic effects and reproduce typical AD pathology in COs. These results confirm our hypothesis that T2DM can accelerate the AD pathological cascade, providing important support for the Aβ cascade hypothesis. The sAD modelling approach developed in this study may offer not only a new strategy for creating clinically relevant sAD models in NHP, but also, when combined with microfluidic organ‐on‐chip technology, could enable the development of a sAD CO model. This integrated platform would provide a valuable high‐throughput tool for investigating AD pathogenesis and screening potential new drug targets.

## 4 Methods

### 4.1 Preparation of Injection Solutions

A 100% solution of 1,1,1,3,3,3‐hexafluoro‐2‐propanol (HFIP, Sigma) was chilled, and 1 mg each of Aβ42, hIAPP, and scrambled Aβ42 (SAβ) lyophilized peptide powder was dissolved separately using a Hamilton syringe. A colorless and transparent peptide solution (1 mm concentration) was obtained by sonication followed by 1 h incubation in a water bath. A transparent peptide film was formed by HFIP removal using a nitrogen stream, during which approximately 30%–50% of the peptide was lost. The peptides were resuspended by adding 20 µL of DMSO. The peptide film was sonicated in an ice‐water bath to ensure complete dissolution, then diluted to 1 mL with cold 10 mM HEPES. Protein concentration was measured using the BCA assay. Aβ42 and SAβ were stored at 4°C for 24 h before use (during which Aβ42 monomers aggregate to form AβO). Based on concentration measurements, appropriate amounts of Aβ42 and hIAPP were mixed at a 1:1 mass ratio to obtain Aβ42‐hIAPP co‐oligomers. The peptide solutions were freshly prepared before each injection and characterized by western blot.

### 4.2 Molecular Docking

Search for the protein on the AlphaFold database, filtering for suitable species, chain length, and resolution, then download the IAPP PDB file. Since the conformational flexibility of Aβ42 did not allow for a high‐resolution crystal structure, a homology modeling strategy was used to construct an initial model using SWISS‐MODEL, and a short‐range simulation of 10 ns was performed by GROMACS to screen out the most stable conformations as docking inputs. Next, utilize the Gramm docking platform for protein interaction prediction to generate candidate conformations. Screen these based on binding energy and conformational rationality to select the optimal conformation. Upload the PDB file of the best conformation to the PDBePISA website to predict protein interaction forces, amino acid residues, interaction surface area, binding energy, and other data. Finally, based on the PDBePISA analysis results, use PyMOL for visualization. Differentiate hydrogen bonds, salt bridges, and other interaction types by color, and output the visualization file.

### 4.3 Molecular Dynamics Simulation

The protein complex obtained from molecular docking is to be further used in molecular dynamics simulation calculations, utilizing GROMACS (2020.3‐MODIFIED) to create the topology file for the protein, with the force field selected as amber99sb‐ildn.ff. Before the molecular dynamics simulations, all systems comprising ions, solvent, receptors, and ligands underwent a thorough preparatory phase, divided into three main steps. Initially, energy minimization was carried out using the steepest descent algorithm, consisting of 5000 steps. This process is aimed at stabilizing the system. Subsequently, during the heating phase to 300 K, positional restraints were applied to the receptors and ligands in each system for a duration of 100 ps. The NVT (Number of atoms, Volume, Temperature) ensemble was employed, using the leap‐frog integrator, with a simulation time step of 100 ps to maintain structural stability. The third stage involved the application of the NPT (Number of atoms, Pressure, Temperature) ensemble, maintaining a constant pressure of 1 pa and a temperature of 300 K. This NPT equilibration phase also lasted for 100 ps. Following these preparatory steps, an unrestrained molecular dynamics simulation was conducted for 100 ns. After completing the 100 ns MD simulation, various dynamic analyses were performed using GROMACS's in‐built scripts, including calculations of Root Mean Square Deviation (RMSD), Root Mean Square Fluctuation (RMSF), Radius of Gyration (Rg), and the number of hydrogen bonds analysis.

### 4.4 Transmission Electron Microscopy (TEM) Analysis

The prepared Aβ42‐hIAPP co‐oligomer peptide solution, AβO peptide solution and scrambled Aβ42 peptide solution were diluted to 0.05 mg/mL and filtered through a 0.22 µm membrane. A 10 µL aliquot of each solution was applied dropwise onto carbon‐coated copper grids and negatively stained with 10 µL of 1% phosphotungstic acid aqueous solution. The samples were then completely dried under a mild fluorescent lamp. TEM observation and image recording were subsequently performed.

### 4.5 Kinetic Thioflavin‐T (ThT) Assay

Aggregation kinetics of scrambled Aβ, hIAPP, Aβ42, and Aβ42‐hIAPP co‐oligomer were monitored using ThT fluorescence, with a blank control containing only HEPES buffer and ThT (Sigma Aldrich, USA). Protein concentrations were 10 µm, and each well contained 20 µL protein solution mixed with 80 µL of 6 µm ThT in black‐clear bottom 96‐well plates. Samples were incubated at RT for 24 h in a SpectraMax iD3 plate reader. The fluorescence was measured at 450 nm excitation and 490 nm emission. All experiments were performed in triplicate and analyzed using GraphPad Prism 9.4.0.

### 4.6 Cell Real‐Time Analysis

1.5 × 104 SH‐SY5Y cells were inoculated in a culture plate and placed in the loading tank of the smart cell real‐time monitor (CM100‐α, East China University of Science and Technology, Shanghai, six broad beans). After cell attachment, CON, AβO, and Aβ42‐hIAPP co‐oligomers were added, respectively, for incubation treatment, and cell status was continuously monitored for up to 96 h. SH‐SY5Y [SHSY‐5Y] (Procell CL‐0208) were kindly provided by *Procell Life Science&Technology Co., Ltd*.

### 4.7 Western Blot Analysis

The Aβ content in Aβ42‐hIAPP co‐oligomers, AβO, and Scrambled Aβ42 was analyzed using 15% SDS‐glycine‐PAGE. The proteins were transferred to a membrane for 7 min at 15 V using an iBlot 2 dry blotting system. The membrane was then blocked by shaking at room temperature in 5% skimmed milk powder for 2 h. Following blocking, the membrane was probed with primary antibodies and incubated overnight at 4°C, and then incubated with secondary antibodies for 1 h at RT. Chemiluminescence was detected using an ECL reagent, and images were captured using the imaging system.

### 4.8 In Vitro Fluorescent Labelling of Peptides

A 1 mg aliquot of IRB‐NHS fluorescent probe was dissolved in 100 µL of DMSO and thoroughly mixed to obtain a 100 µg/mL stock solution. Add 200 µL of peptide solution (0.5 mg/mL) to 20 µL of fluorescent labelling reagent solution and incubate at RT for 2 h. After the reaction, the mixture was transferred to a 3 kd ultrafiltration tube and centrifuged three times to remove the unbound fluorescent probe and concentrate the peptide. Mixtures were subsequently transferred to 3 kDa ultrafiltration tubes, which were centrifuged to 100 µL residual volume to remove free fluorescent probe and concentrate the peptide. The resulting green‐colored peptide solution at 1 mg/mL concentration was directly used for COs injection.

### 4.9 Generation of COs

The COs used in this study were derived from a single iPSC line [69] provided by iRegene Therapeutics Co., Ltd. Cerebral organoids were prepared according to a previously established protocol [70]. Briefly, iPSCs were dissociated with Accutase (STEMCELL Technologies, 07920) to obtain the single‐cell suspensions, and seeded in AggreWell800 24‐well Plate (STEMCELL, 34811) to form embryoid bodies (EBs) following the manufacturer's protocol. The following day, the medium was switched to Brain Organoid Induction Medium (iRegene Therapeutics, RJ3001A), with half of the medium being replaced every other day. On day 5, organoids were transferred to an ultra‐low attachment plate and cultured on an orbital shaker (80 rpm). From day 10 onward, organoids were maintained in Organoid Maturation Medium (iRegene Therapeutics, RJ3001B) with half‐medium changes every two days.

### 4.10 Peptide Injection into the Center of COs

The Nanoject III syringe pump was attached to the stereotaxic system. Trim the tip of the capillary needle (outer diameter ∼40 µm) and attach it to the injection pump's capillary holder. Use the capillary to aspirate 2 µL of the fluorescently labelled peptide solution. Using the stereotaxic system, align the needle with the center of the CO and advance it slowly at 1 mm/min to a depth of 1 mm. Slowly inject 1 µL of the peptide solution into the center of the CO (injection rate: 5 nL/s). After injection, withdraw the needle slowly at the same speed. Each COs injection is performed according to standard operating procedures. The COs are examined under a fluorescence microscope to confirm successful injection and observe the dynamics of the injected peptide within the COs. Four COs were injected in each of the Aβ42‐hIAPP co‐oligomer, AβO, SAβ, and HEPES groups.

### 4.11 COs sample preparation

COs were transferred to 4% paraformaldehyde for fixation and then overnight at 4°C. The COs were sugar‐sedimented with 30% sucrose solution at RT for 1 h. After the COs were bottomed out, they were transferred to embedding cassettes filled with fish gelatin. The tissues were equilibrated at RT for 30 min and then placed at −80°C for quick freezing. COs were sliced into 12 µm using a cryosectioner and stored at −80°C. Based on the total number of CO sections, we divided them into 6–8 layers according to the principle of central symmetry (Figure S1D). Sections were selected for pathological characterization in accordance with this symmetry principle.

### 4.12 Immunohistochemical Analysis

Briefly, antigen retrieval was performed using citrate buffer (pH 6.0). Tissue sections were incubated with primary antibody (Table S5) at 4°C for 16 h, followed by a 2 h of incubation with horseradish peroxidase (HRP)‐conjugated secondary antibody. Immune complexes were visualized using the avidin‐biotin‐peroxidase complex (prepared from the VECTASTAIN ABC Reagent kit) and the chromogenic substrate 3,3’‐diaminobenzidine tetrahydrochloride hydrate (DAB). Images were acquired using a VS200 Virtual Slide Microscope (Olympus, Japan).

### 4.13 Immunofluorescence Analysis

Briefly, primary antibodies raised in different host species were incubated with samples at 4°C overnight. This was followed by a 1 h incubation at room temperature with species‐matched secondary antibodies conjugated to Alexa Fluor 488, Alexa Fluor 594, or Alexa Fluor 647. To preserve fluorescence, samples were mounted with an anti‐fade mounting medium containing DAPI. Immunofluorescence images were acquired using a Nikon acquired confocal microscope.

### 4.14 TUNEL Assay

Organoid slices were permeabilized with 0.3% Triton X‐100 for five minutes at RT after being cleaned three times with PBS. After that, add 50 µL of TUNEL reagent, incubate for 60 min at 37°C, and then wash with PBS. DAPI was used to counterstain nuclei for 5 min. The images were captured using a fluorescence microscope.

### 4.15 Thioflavin S Staining

Sections of 12 µm COs were immersed in 0.025% thioflavin S (solubilized in 50% ethanol) and incubated at room temperature for 30 min. The sections were decolorized in 50% ethanol, co‐stained with DAPI, and sealed with an antifade mounting medium. Images were scanned with an Olympus BX53 microscope.

### 4.16 Congo Red Staining

Sections of 12 µm COs were stained in Congo red staining solution (Congo red 0.5 g, methanol 80 mL, glycerol 20 mL) for 20 min, and then put into alkaline ethanol differentiation solution (potassium hydroxide 0.2 g, 80% ethanol 100 mL). Finally, the sections were stained with hematoxylin and sealed with neutral resin. Slides were scanned by a scanning microscopy imaging system (Olympus, Japan).

### 4.17 Capillary‐Based Immunoassays

COs tissue was lysed using cell lysis buffer (Beyotime, P0013), and the supernatant was collected following centrifugation. Protein expression and phosphorylation levels were quantified using the Wes automated Western blotting system (ProteinSimple, USA) in strict accordance with the manufacturer's protocol. Cell lysates and reagents were loaded into assay wells, allowing the capillary system to automatically aspirate and bind the liquid‐phase primary antibody to target proteins. Immunodetection was carried out using an HRP‐conjugated secondary antibody and a chemiluminescent substrate. Fluorescent signals were detected by the WES imaging system and quantified with Compass for SW software.

### 4.18 Metabolomic Analysis

The metabolomics of COs cultures at D0, D15, D45, and D90 were analyzed. An LC‐ESI‐MS/MS system (UPLC, ExionLC, AD https://sciex.com.cn/; MS, QTRAP system, https://sciex.com/) from Wuhan Metware Biotechnology Co., and mass spectrometry analysis was performed by ultra‐performance liquid chromatography and tandem mass spectrometry. The mass spectrometry data were processed using the software Analyst 1.6.3. PCA was performed using the statistical function prcomp within R (www.rproject.org). Differential metabolites that were significantly regulated between groups were screened by VIP > 1 and *p*‐value < 0.05, and FC value = 1.2 (Student's t test) and analyzed for KEGG enrichment.

### 4.19 MTT Cell Viability Assay

After the cells have been exposed to the peptide solution for the specified duration, add 10 µL of MTT solution at a concentration of 5 mg/mL to each well. Continue incubation at 37°C for 4 h. After adding 100 µL of formazan solution to each well, continue incubation at 37°C. The optical density (OD) values were measured at 570 nm using a microplate reader (AMR‐100, China). Each group contained three replicate samples.

### 4.20 Statistical Analysis

Data are expressed as mean ± SD. For comparing multiple groups, a one‐way ANOVA with post hoc Tukey's test was used. Unpaired Student's *t*‐test (two‐tailed) was used to compare two groups. Significant p values are defined as ns *p* > 0.05, ^*^ *p* ≤ 0.05, ^**^ *p* ≤ 0.01, ^***^ *p* ≤ 0.001, and ^****^ *p* ≤ 0.0001. Correlation analysis employed Pearson's correlation coefficient. Statistical analysis was carried out using GraphPad Prism 9.5.1 (GraphPad, San Diego, CA, USA). Statistical details of each experiment can be found in the figure legends.

## Author Contributions

J.Y. was responsible for figuring out experimental protocols, carrying out experiments, analyzing data, drawing conclusions and writing manuscript. Z.T. and Y.L. performed experiments; B.L. provided experimental method support and calculation analysis; X.H. and J.W. involved in figuring out experimental protocols. F.Y. conceived the original idea, supervised the study, reviewed the manuscript, and provided funding support. All authors have approved the final version of the manuscript, including the order of authorship.

## Funding

The STI2030‐Major Projects (2021ZD0200900) and Project of Collaborative Innovation Center of One Health (XTCX2022JKC05).

## Conflicts of Interest

All authors declare no conflicts of interest.

## Supporting information




**Supporting file**: advs73970‐sup‐0001‐SuppMat.docx

## Data Availability

Data available on request from the authors.
