# Supplementary material for: Islet Amyloid Polypeptide Modelled to Simulate Diabetes Co‐Oligomerized with β‐Amyloid 1‐42 Reproducing the Pathological Cascade of Alzheimer's Disease in Human Cerebral Organoids
Source: Adv Sci (Weinh). 2026 Jan 22;13(18):e16837. doi: 10.1002/advs.202516837 (PMC13042975; doi:10.1002/advs.202516837)
Supplement: Supplementary file 1 — Supporting file: advs73970‐sup‐0001‐SuppMat.docx [file ADVS-13-e16837-s001.docx]

**Supporting Information:**

**Islet Amyloid Polypeptide Modelled to Simulate Diabetes Co-Oligomerized with β-Amyloid 1-42 Reproducing the Pathological Cascade of Alzheimer's Disease in Human Cerebral Organoids**

*Jin Yan^1,2^, Zhimeng Tang^1,2^, Yanyu Luo^1,2^, Bin Liu^1,2^, Xinxin Huang^1,2^, Jun Wei^3^, Feng Yue^1,2,^ **

^1^State key Laboratory of Digital Medical Engineering, School of Biomedical Engineering, Hainan University. Sanya, 572025, China.

^2^Collaborative Innovation Center of One Health, Hainan University. Haikou 570228, China.

^3^iRegene Therapeutics Co. Ltd., Chengdu 610200, China.

*Corresponding author. Email: fyuee@hotmail.com (F.Y.)


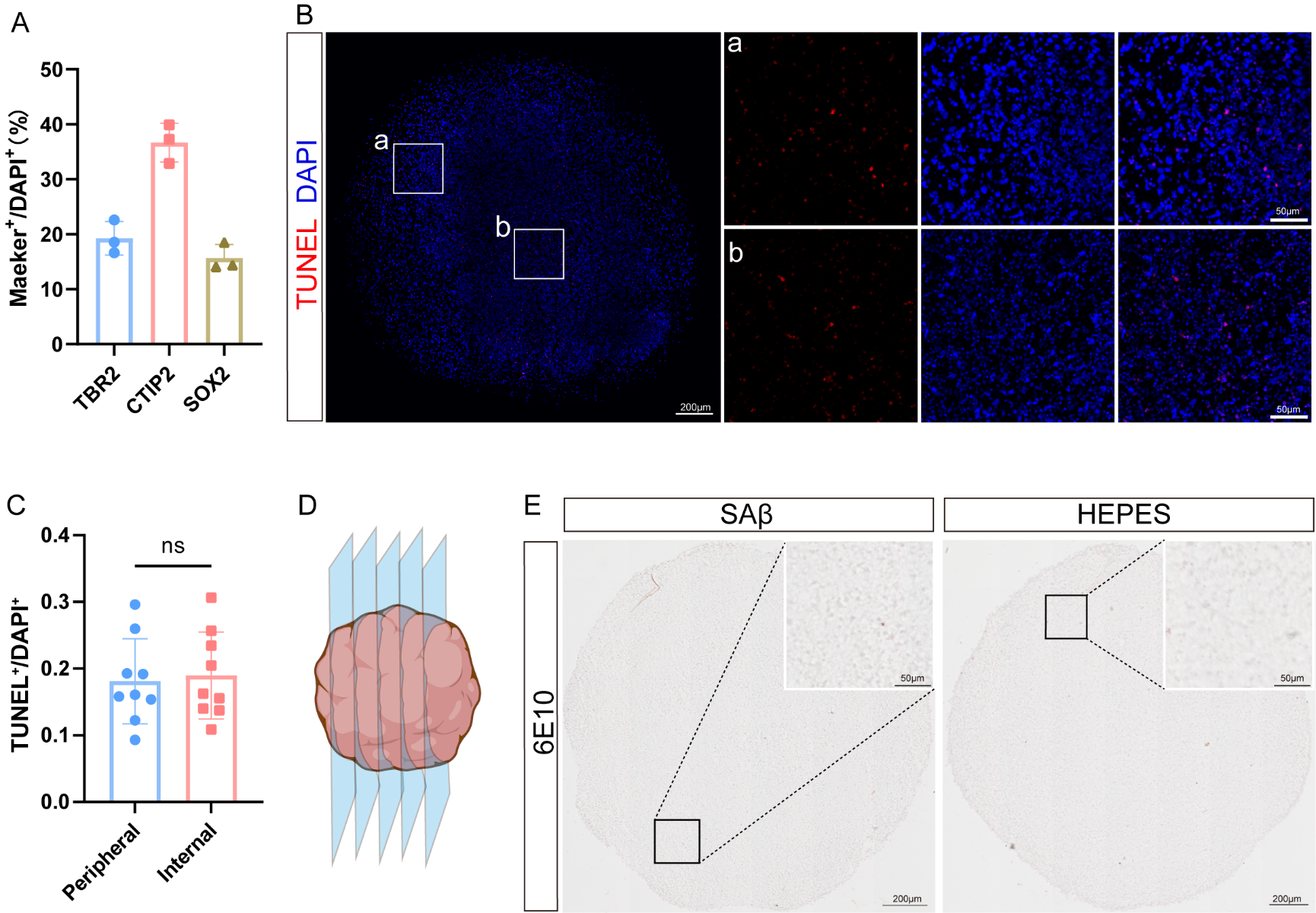


**Figure S1. Histological staining of Cerebral Organoids (CO).**

A. Quantitative analysis of the percentage of SOX2-, TBR2- and CTIP2-positive cells in mature COs. Data are represented as mean ± SD (n = 3 CO slices for each marker).

B. Representative TUNEL staining images of mature D100 organoids. a: internal regions, b: peripheral regions. Scale bar: 200μm, 50 μm.

C. Quantification of TUNEL+/DAPI+ cells in the internal and peripheral regions of COs. 9 images (20×) were analyzed in both the internal and peripheral regions.

D. The schematic diagram shows 6–8 layers of the CO from anterior to posterior for pathological characterization.

E. Immunohistochemical analysis using 6E10 antibody for detection of Aβ plaque production in COs of SAβ and HEPES groups. Related to Figure 3. Scale bar: 200μm, 50 μm.

Data are represented as mean ± SD. Unpaired two-tailed t-test was used to compare mean difference between each group.


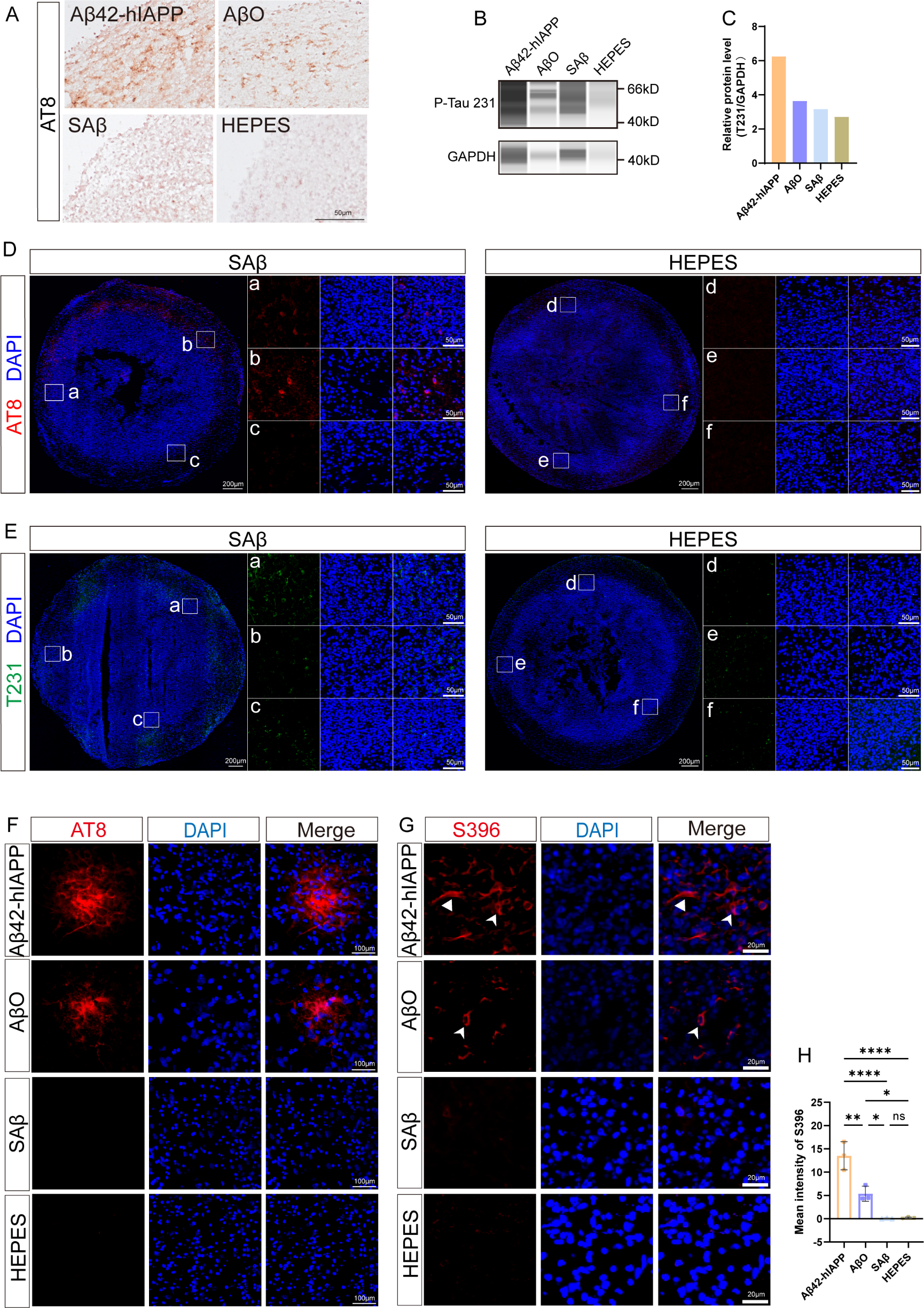


**Figure S2. Histological staining of NFT pathological features in COs induced by Aβ42-hIAPP co-oligomer, AβO, SAβ and HEPES.** Related to Figure 4.

A. Immunohistochemical staining characterized AT8 levels in COs of the Aβ42-hIAPP co-oligomer, AβO, SAβ and HEPES groups. Scale bar: 50 μm.

B-C. Capillary-based immunoassays of p-tau (T231) in COs of Aβ42-hIAPP co-oligomers, AβO, SAβ and HEPES group (B), and normalized to GAPDH (C).

D. Immunofluorescence staining images of AT8 in COs of SAβ and HEPES groups. Scale bar: 200 μm, 50 μm.

E. Immunofluorescence staining images of T231 in COs of SAβ and HEPES groups. Scale bar: 200 μm, 50 μm.

F. COs of Aβ42-hIAPP co-oligomer and AβO groups contained AT8 immunofluorescence-positive neuritic plaques. Scale bar: 100 μm.

G-H. Immunofluorescence staining (G) and mean intensity quantification (H) p-tau (S396) in COs of Aβ42-hIAPP co-oligomers, AβO, SAβ, and HEPES groups. 3 tissue sections were randomly selected for each group. Scale bar: 20 μm.

Data are represented as mean ± SD. n=6 independent slices for each group (H). P-values are calculated using One-way ANOVA testing followed by a Tukey post-hoc test (H). ns p＞0.05, *p ≤ 0.05; **p ≤ 0.01; ****p ≤ 0.0001.


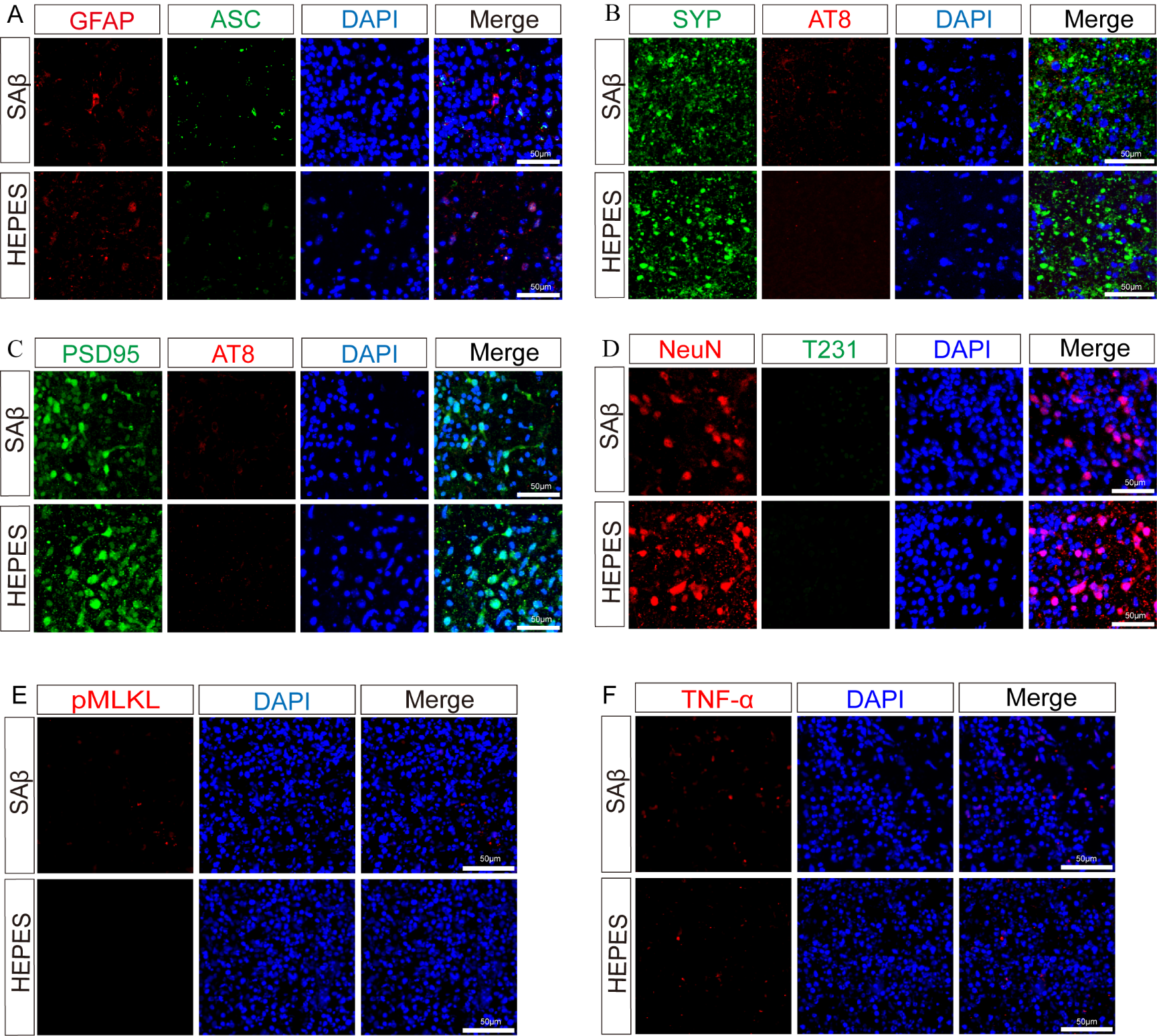


**Figure S3. Characteristic pathology of AD in COs of the SAβ and HEPES groups.** Related to Figures 5-7.

A. Merge image of astrocyte (GFAP) immunofluorescence staining (red) and ASC (green) in COs of SAβ and HEPES groups. Scale bar: 50 μm.

B. Merge image of p-tau (AT8) immunofluorescence staining (red) and SYP (green) in COs of SAβ and HEPES groups. Scale bar: 50 μm.

C. Merge image of p-tau (AT8) immunofluorescence staining (red) and PSD95 (green) in COs of SAβ and HEPES groups. Scale bar: 50 μm.

D. Merge image of p-tau (T231) immunofluorescence staining (green) and NeuN (red) in COs of SAβ and HEPES groups. Scale bar: 50 μm.

E. Immunofluorescence staining images of necrotic apoptotic markers (pMLKL) in COs of SAβ and HEPES groups. Scale bar: 50 μm.

F. Immunofluorescence staining images of inflammatory factors (TNF-α) in COs of SAβ and HEPES groups. Scale bar: 50 μm.


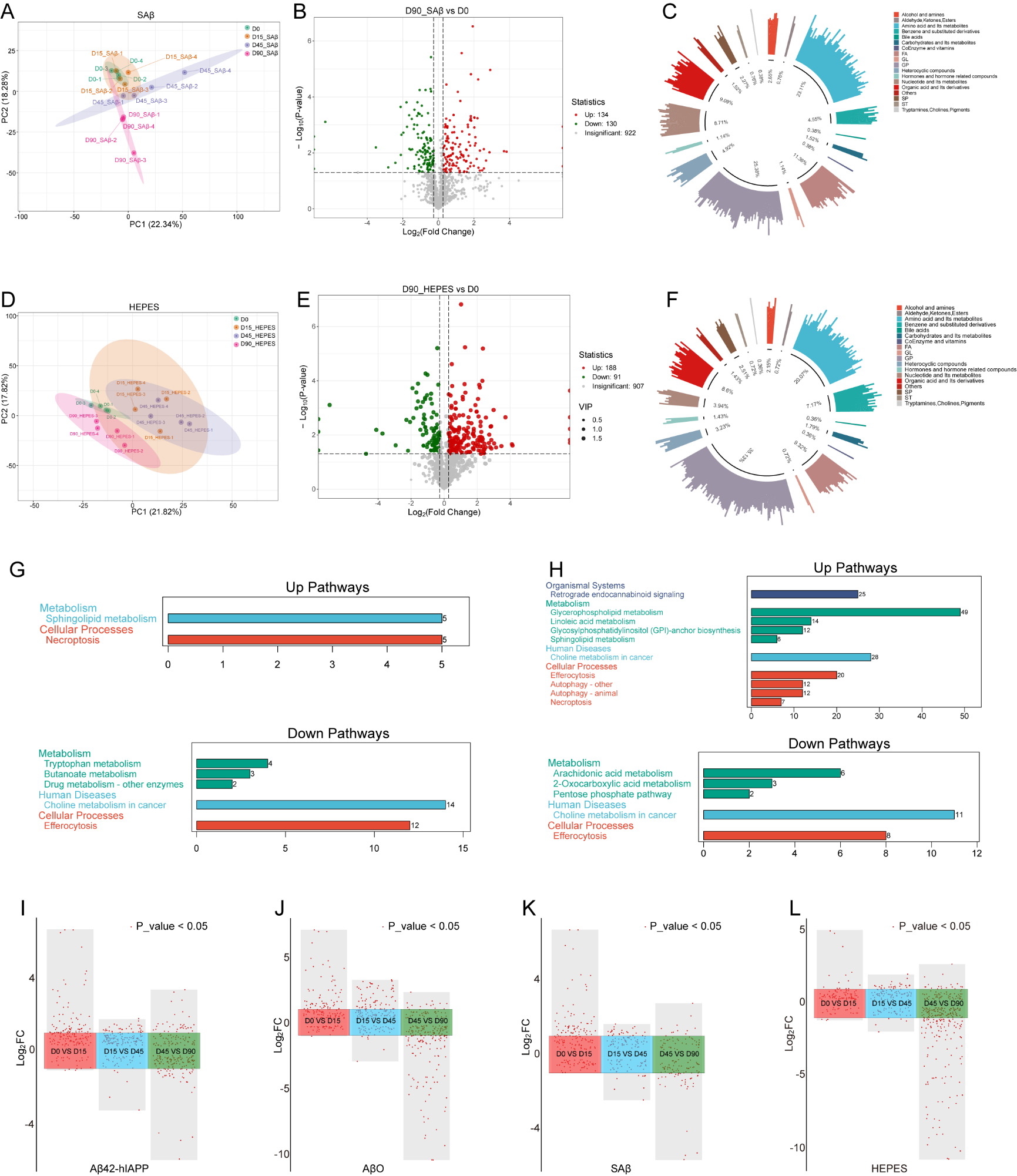


**Figure S4. Metabolomic analysis for COs cultures of SAβ and HEPES groups during D0-D90.** Related to Figure 8.

A, D. PCA plots of SAβ (A) and HEPES group (D) at D0, D15, D45, and D90. Points marked with the same colour represent four replicates of each group.

B, E. Volcano plots show the metabolite differences that appeared during D0-D90 in the SAβ group (B) and HEPES group (E). Screening of differentially expressed metabolites required both Fold change (FC) > 1.2, VIP > 1, and P-value < 0.05 (Student's test). Green spots indicate down-regulated differentially expressed metabolites; red spots indicate up-regulated differentially expressed metabolites; and gray spots indicate metabolites with insignificant differences detected. X axis: FC; y axis: log10 (P-values).

C, F. Plots of occupied circles for the first-level classification of SAβ group (C) and HEPES group (F), showing the categorization information of the differential metabolites. The colors of the outer circles and the length of the columns represent the different classes of substances and their relative content levels. The inner circle is the ratio of the amount of each class of substance to the amount of all substances.

G-H. Significantly up and down regulated KEGG metabolic pathways enriched in the SAβ group (G) and HEPES group (H) during D0-D90. Y-axis: Significantly enriched pathways across different KEGG Level 1 classifications. X-axis: Number of DEMs enriched into each pathway.

I-L. Volcano plots showing differential metabolites appearing in dynamic metabolomics (D0-D15, D15-D45, D45-D90) for the Aβ42-hIAPP co-oligomer (I), AβO (J), SAβ (K), and HEPES (L) groups, respectively. The y-axis corresponds to log2 ((FC).

N=4 biologically independent samples in each group.


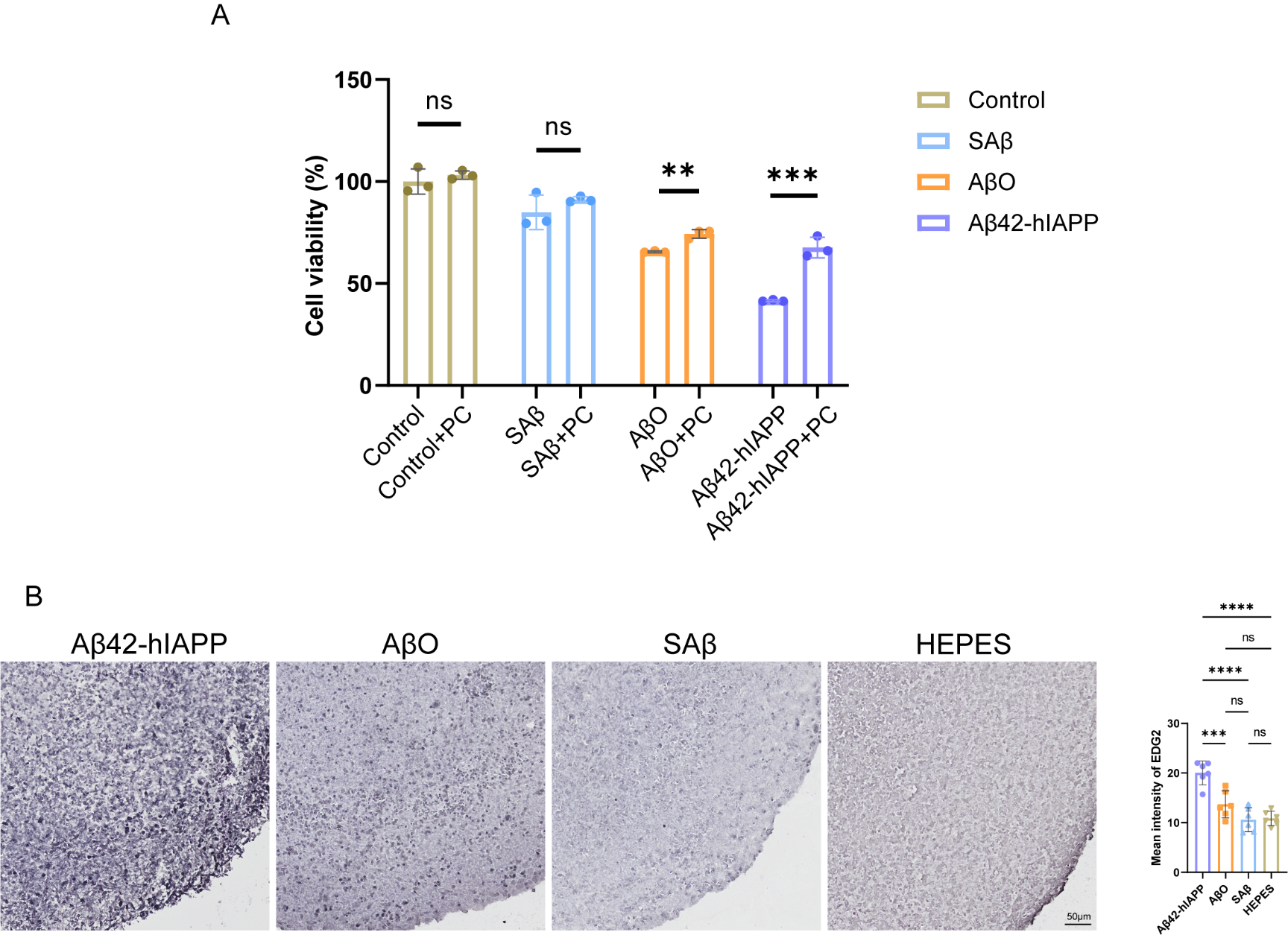


**Figure S5. Supplementary validation and targeted validation of DEM.**

A. SH-SY5Y cells were treated with 3 μg/mL PC for 24 hours. Subsequently, 10 μM SAβ, AβO, and Aβ42-hIAPP oligomers was applied to the cells for 48 hours, respectively. Cell viability was assessed using the MTT assay.

B. Histological staining of EDG2 receptors in COs induced by Aβ42-hIAPP co-oligomer, AβO, SAβ and HEPES. Scale bar: 50 μm.

Data are represented as mean ± SD. n = 3 for each group, and unpaired t-test was applied (A). n=6 independent slices for each group, and p-values are calculated using One-way ANOVA testing followed by a Tukey post-hoc test (B). ns p＞0.05, **p ≤ 0.01, ***p ≤ 0.001; ****p ≤ 0.0001.

**Table S1. Correlations between Aβ plaques and tau pathology, synaptic injury, neuroinflammation, and neuronal injury pathology.**

| Correlation | Pearson R | *P Value* |
| --- | --- | --- |
| 6E10-T231 | 0.6797 | **0.0106** |
| 6E10-AT8 | 0.6423 | **0.0179** |
| 6E10-SYP | -0.6643 | **0.0133** |
| 6E10-PSD95 | -0.6617 | **0.0138** |
| 6E10-GFAP | 0.7506 | **0.0031** |
| 6E10-ASC | 0.7952 | **0.0012** |
| 6E10-NeuN | -0.6467 | **0.0169** |

A total of 16 slices were randomly selected for correlation analysis. Data were analysed using Pearson correlation in GraphPad Prism.

**Table S2. Correlation between tau pathology and synaptic injury, neuroinflammation, and neuronal damage.**

| Correlation | Pearson r | **P Value** |
| --- | --- | --- |
| T231-SYP | -0.7654 | **0.0005** |
| AT8-SYP | -0.8893 | **＜0.0001** |
| T231-PSD95 | -0.8189 | **0.0001** |
| AT8-PSD95 | -0.9368 | **＜0.0001** |
| T231-GFAP | 0.9244 | **＜0.0001** |
| AT8-GFAP | 0.9181 | **＜0.0001** |
| T231-ASC | 0.8864 | **＜0.0001** |
| AT8-ASC | 0.7841 | **0.0003** |
| T231-NeuN | -0.8518 | **＜0.0001** |
| AT8-NeuN | -0.857 | **＜0.0001** |

A total of 16 slices were randomly selected for correlation analysis. Data were analysed using Pearson correlation in GraphPad Prism.

**Table S3. Correlation analysis of immunofluorescence co-staining results in COs.**

| Group | Correlation | Pearson r | **P Value** |
| --- | --- | --- | --- |
| Aβ42-hIAPP | GFAP-ASC | 0.8414 | **＜0.01** |
|  | AT8-SYP | -0.7339 | **＜0.05** |
|  | AT8-PSD95 | -0.7932 | **＜0.01** |
|  | NEUN-T231 | -0.7383 | **＜0.01** |
| AβO | GFAP-ASC | 0.7292 | **＜0.01** |
|  | AT8-SYP | -0.6967 | **＜0.05** |
|  | AT8-PSD95 | -0.7729 | **＜0.01** |
|  | NEUN-T231 | 0.6670 | **＜0.05** |
| SAβ | GFAP-ASC | 0.7227 | **＜0.01** |
|  | AT8-SYP | -0.6178 | **＜0.05** |
|  | AT8-PSD95 | -0.6124 | **＜0.05** |
|  | NEUN-T231 | -0.6202 | **＜0.05** |
| HEPES | GFAP-ASC | 0.6593 | **＜0.05** |
|  | AT8-SYP | -0.6122 | **＜0.05** |
|  | AT8-PSD95 | -0.6120 | **＜0.05** |
|  | NEUN-T231 | -0.6084 | **＜0.05** |

n=4 COs for each of the Aβ42-hIAPP co-oligomers, AβO, SAβ and HEPES groups, 3 images (20×) are randomly selected for each CO. Data were analysed using Pearson correlation in GraphPad Prism.

**Table S4. Dynamics of differential KEGG metabolic pathways in COs-induced cultures at three incubation time periods (during D0-D15, D15-D45, and D45-D90).**

| Time Period | Aβ42-hIAPP | AβO | SAβ | HEPES | Type |
| --- | --- | --- | --- | --- | --- |
| Dynamic metabolomics  D0-D15 | - Retrograde endocannabinoid signaling - Glycerophospholipid metabolism - Glycosylphosphatidylinositol (GPI)-anchor biosynthesis - Autophagy - animal - Autophagy - other - Kaposi sarcoma-associated herpesvirus infection - Pathogenic Escherichia coli infection - Linoleic acid metabolism - *alpha-Linolenic acid metabolism* - Arachidonic acid metabolism - Choline metabolism in cancer - Metabolic pathways | - Glycosylphosphatidylinositol (GPI)-anchor biosynthesis - Autophagy - animal - Autophagy - other - Kaposi sarcoma-associated herpesvirus infection - Pathogenic Escherichia coli infection | - Retrograde endocannabinoid signaling - Glycosylphosphatidylinositol (GPI)-anchor biosynthesis - Autophagy - animal - Autophagy - other - Kaposi sarcoma-associated herpesvirus infection - Pathogenic Escherichia coli infection - Primary bile acid biosynthesis - Bile secretion - Glycerophospholipid metabolism - Riboflavin metabolism | - Bile secretion - Folate biosynthesis - Primary bile acid biosynthesis - Riboflavin metabolism - Protein digestion and absorption - 2-Oxocarboxylic acid metabolism - Mineral absorption - Biosynthesis of cofactors | UP |
|  | - Drug metabolism - other enzymes - Riboflavin metabolism | - Biosynthesis of cofactors - Nicotinate and nicotinamide metabolism - Drug metabolism - other enzymes - Prolactin signaling pathway | - Nicotinate and nicotinamide metabolism - Propanoate metabolism | - Prolactin signaling pathway - Riboflavin metabolism - Ubiquinone and other terpenoid-quinone biosynthesis - Melanogenesis - Cocaine addiction - Amphetamine addiction - Alcoholism - Biosynthesis of cofactors - Thiamine metabolism - Dopaminergic synapse - Parkinson disease - Phenylalanine, tyrosine and tryptophan biosynthesis | Down |
| Dynamic metabolomics  D15-45 | - Efferocytosis - Choline metabolism in cancer | - Nucleotide metabolism - Taurine and hypotaurine metabolism - Sphingolipid signaling pathway | - Glycerophospholipid metabolism - Choline metabolism in cancer - Retrograde endocannabinoid signaling - Glycosylphosphatidylinositol (GPI)-anchor biosynthesis - Autophagy - animal - Autophagy - other - Kaposi sarcoma-associated herpesvirus infection - Pathogenic Escherichia coli infection - Efferocytosis - Fc gamma R-mediated phagocytosis - GnRH signaling pathway - Pancreatic cancer | - | UP |
|  | - *Glycine, serine and threonine metabolism* - Bile secretion | - Arachidonic acid metabolism | - Biosynthesis of cofactors - Pyrimidine metabolism - One carbon pool by folate | - Pyrimidine metabolism - Cholesterol metabolism | Down |
| Dynamic metabolomics  D45-D90 | - *Neuroactive ligand-receptor interaction* - Choline metabolism in cancer - Primary bile acid biosynthesis - Necroptosis - Arachidonic acid metabolism | - Necroptosis - Efferocytosis - Sphingolipid metabolism - Sphingolipid signaling pathway - Glycerophospholipid metabolism - *alpha-Linolenic acid metabolism* - Linoleic acid metabolism | - Efferocytosis | - Metabolism of xenobiotics by cytochrome P450 | UP |
|  | - Choline metabolism in cancer - Efferocytosis - *Caffeine metabolism* - Nucleotide metabolism - Tryptophan metabolism | - Nucleotide metabolism - Tryptophan metabolism - Purine metabolism - Cysteine and methionine metabolism - Biosynthesis of amino acids | - Efferocytosis - Choline metabolism in cancer - Mineral absorption | - Purine metabolism - Nucleotide metabolism - Tryptophan metabolism - Bile secretion - Ether lipid metabolism - cGMP-PKG signaling pathway - Morphine addiction - Renin secretion - African trypanosomiasis - Efferocytosis - Choline metabolism in cancer | Down |

All KEGG pathways P value＜0.05.

**Table S5. KEGG pathways common to Aβ42-hIAPP and AβO group in systemic (D0-D90) and dynamic metabolomics (D0-D15; D15-D45; D45-D90).**

| Time Period | Description | Class Ⅱ | Compounds |
| --- | --- | --- | --- |
| Systematic metabolomics (D0-D90) of the co-KEGG pathway in the Aβ42-hIAPP and AβO groups | Arachidonic acid metabolism（UP） | Oxidized lipids | 20-carboxy LTB4 |
|  |  | PC | PC(14:0_14:0); PC(16:0_16:0); PC(16:0_18:1); PC(14:1_16:1); PC(15:0_16:0); PC(14:0_16:1); PC(17:0_18:1); PC(14:0_18:2); PC(16:0_18:3); PC(18:2_22:1); PC(18:0_22:4) |
|  |  | PC-O | PC(O-16:0_14:0); PC(O-18:0_16:0); PC(O-18:1_16:0); PC(O-16:1_18:2); PC(O-18:1_20:4) |
|  | alpha-Linolenic acid metabolism（UP） | PC | PC(14:0_14:0); PC(16:0_16:0); PC(16:0_18:1); PC(14:1_16:1); PC(15:0_16:0); PC(14:0_16:1); PC(17:0_18:1); PC(14:0_18:2); PC(16:0_18:3); PC(18:2_22:1); PC(18:0_22:4); |
|  |  | PC-O | PC(O-16:0_14:0); PC(O-18:0_16:0); PC(O-18:1_16:0); PC(O-16:1_18:2); PC(O-18:1_20:4) |
| Dynamic metabolomics of the co-KEGG pathway in the Aβ42-hIAPP (D0-D15) and AβO (D45-D90) groups | alpha-Linolenic acid metabolism（UP） | PC | PC(16:0_18:1); PC(17:0_18:1) |

All KEGG pathways P value＜0.05

**Table S6. KEGG pathway unique to the Aβ42-hIAPP group in systemic (D0-D90) and dynamic metabolomics (D0-D15; D15-D45; D45-D90).**

| Time Period | Description | Class Ⅱ | Compounds |
| --- | --- | --- | --- |
| Systematic metabolomics  (D0-D90) | Glycine, serine and threonine metabolism（down） | PS | PS (14:0_19:0); PS (18:0_22:3); PS (14:0_21:0); PS (16:0_19:0); PS (16:0_21:0); PS (21:0_18:1) |
|  |  | Cholines | Choline |
|  |  | Amino acids | L-Tryptophan |
|  |  | Others | Betaine |
| Systematic metabolomics  (D0-D90) | Chemical carcinogenesis - reactive oxygen specie（down） | Amino acid derivatives | S-(5-Adenosy)-L-Homocysteine |
| Dynamic metabolomics  (D0-D15) | Metabolic pathways（up） | Alcohols | 9-Fluorenol; Bis(1-inositol)-3,1'-phosphate 1-phosphate |
|  |  | Amino acid derivatives | N-acetyl-D-phenylalanine |
|  |  | Amino acids | L-Valine |
|  |  | BA | Glycocholic acid |
|  |  | Benzene and substituted derivatives | 2-Phenylacetamide; 2-(4-Hydroxyphenyl) ethanol |
|  |  | CoEnzyme and vitamins | Riboflavin |
|  |  | FFA | FFA (18:2) |
|  |  | Heterocyclic compounds | 4-Amino-5-hydroxymethyl-2-methylpyrimidine |
|  |  | HexCer-NS | HexCer (d18:1/18:0) |
|  |  | Indole and Its derivatives | Indole-3-acetamide |
|  |  | LPC-O | LPC(O-18:0) |
|  |  | Organic acid and Its derivatives | 3-Hydroxycinnamic acid; 4-Guanidinobutyric Acid;  Salicylic acid |
|  |  | PA | PA (18:0_21:0); PA (20:0_18:1); PA (18:1_20:1) |
|  |  | PC | PC(16:0_16:0); PC(16:0_18:0); PC(16:0_18:1); PC(18:0_18:1); PC(16:0_20:1); PC(18:1_18:1); PC(18:0_18:2); PC(15:1_18:1); PC(17:0_18:1); PC(16:0_22:1); PC(18:0_20:2); PC(18:2_22:1) |
|  |  | PC-O | PC(O-16:0_16:0); PC(O-18:0_16:0); PC(O-18:1_16:0); PC(O-16:0_18:2) |
|  |  | PE | PE (16:0_18:0); PE (18:1_18:0); PE (18:1_18:1); PE (18:0_18:2); PE (20:4_18:0); PE (15:1_22:4) |
|  |  | PE-P | PE(P-16:0_18:1); PE(P-18:0_18:1); PE(P-18:1_18:1); PE(P-18:0_22:3); PE(P-16:0_20:4); PE(P-16:0_16:0); PE(P-16:0_20:2); PE(P-16:0_20:3); PE(P-16:0_22:3); PE(P-16:0_22:4); PE(P-18:1_22:3); PE(P-18:1_22:4) |
|  |  | Phenolic acids | 4-Hydroxybenzoic Acid |
|  |  | Polyamines | Spermidine |
|  |  | PS | PS (18:0_22:3); PS (16:0_21:0); PS (21:0_18:1) |
|  |  | Pteridines and derivatives | N'-Methyl-2-pyridone-5-carboxamide; 5,10-Methylene-THF |
|  |  | SM | SM(d18:0/18:0); SM(d18:1/18:0) |
|  |  | Sugar derivatives | 2,4-diacetamino-2,4,6-triphenoxy-D-mannopyranose |
| Dynamic metabolomics (D115-D145) | Glycine, serine and threonine metabolism（down） | PS | PS (14:0_19:0); PS (18:1_22:4) |
|  |  | Amino acids | Sarcosine |
| Dynamic metabolomics (D145-D190) | Neuroactive ligand-receptor interaction（up） | Sulfonic acids | Taurine |
|  |  | Hormones and hormone related compounds | Epinephrine |
|  |  | Amines | Palmitoylethanolamide (PEA) |
|  |  | LPA | LPA (16:3) |
| Dynamic metabolomics (D145-D190) | Caffeine metabolism（down） | Nucleotide and Its metabolites | Xanthosine; Xanthine; Theobromine |

All KEGG pathways P value＜0.05

**Table S7. Antibodies used in this study.**

| Antibodies | Source | Identifier |
| --- | --- | --- |
| Mouse anti-Aβ_1-16_ (6E10) | Biolegend | Cat #803001 |
| Mouse anti-PHF-tau (Ser202, Thr205) (AT8) | Thermo Fisher Scientific | Cat # MN1020 |
| Anti-Tau (phospho S396) | Abcam | Cat # ab109390 |
| Chicken anti-GFAP | Abcam | Cat # ab4674 |
| Mouse anti-NEUN | Millipore | Cat # MAB377 |
| Rabbit anti-ASC | ABclonal | Cat # ab1170 |
| Rabbit anti-Synaptophysin | Abcam | Cat # ab32127 |
| Rabbit anti-PSD95 | Abcam | Cat # ab18258 |
| Rabbit anti-T231 | Abcam | Cat # ab151559 |
| Anti-MLKL (phospho S358) | Abcam | Cat # ab187091 |
| Rabbit anti-TNF Alpha | Proteintech | Cat # 17590-1-AP; RRID: AB_2271853 |
| Rabbit anti-SATB2 | Abcam | Cat # ab92446 |
| Rabbit anti-SOX2 | Abcam | Cat # ab92494 |
| Goat anti-SOX2 | R&D | Cat # AF2018 |
| Rabbit anti-TBR2 | Abcam | Cat # ab216870 |
| Rat anti-CTIP2 | Abcam | Cat # ab18465 |
| Mouse anti-MAP2 | Gene Tex | Cat # GTX85455 |
| Mouse anti-TUJ1 | Biolegend | Cat # 801202 |
| Goat Anti-Mouse HRP | Servicebio | Cat # GB23301 |
| Goat Anti-Rabbit IgG H&L (Alexa Fluor® 488) | Abcam | Cat # ab150077 |
| Goat Anti-Mouse IgG H&L (Alexa Fluor® 488) | Abcam | Cat # ab150113 |
| Goat Anti-Rabbit IgG H&L (Alexa Fluor® 594) | Abcam | Cat # ab150080 |
| Goat Anti-Mouse IgG H&L (Alexa Fluor® 594) | Abcam | Cat # ab150116 |
| Chicken Anti-Goat IgG H&L (Alexa Fluor® 647) | Abcam | Cat # ab150171 |
